# Host-Detrimental Role of Esx-1-Mediated Inflammasome Activation in Mycobacterial Infection

**DOI:** 10.1371/journal.ppat.1000895

**Published:** 2010-05-06

**Authors:** Fredric Carlsson, Janice Kim, Calin Dumitru, Kai H. Barck, Richard A. D. Carano, Mei Sun, Lauri Diehl, Eric J. Brown

**Affiliations:** 1 Department of Microbial Pathogenesis, Genentech Inc., South San Francisco, California, United States of America; 2 Department of Translational Immunology, Genentech Inc., South San Francisco, California, United States of America; 3 Department of Biomedical Imaging, Genentech Inc., South San Francisco, California, United States of America; 4 Department of Pathology, Genentech Inc., South San Francisco, California, United States of America; University of New Mexico, United States of America

## Abstract

The Esx-1 (type VII) secretion system is a major virulence determinant of pathogenic mycobacteria, including *Mycobacterium marinum*. However, the molecular events and host-pathogen interactions underlying Esx-1-mediated virulence *in vivo* remain unclear. Here we address this problem in a non-lethal mouse model of *M. marinum* infection that allows detailed quantitative analysis of disease progression. *M. marinum* established local infection in mouse tails, with Esx-1-dependent formation of caseating granulomas similar to those formed in human tuberculosis, and bone deterioration reminiscent of skeletal tuberculosis. Analysis of tails infected with wild type or Esx-1-deficient bacteria showed that Esx-1 enhanced generation of proinflammatory cytokines, including the secreted form of IL-1β, suggesting that Esx-1 promotes inflammasome activation *in vivo*. *In vitro* experiments indicated that Esx-1-dependent inflammasome activation required the host NLRP3 and ASC proteins. Infection of wild type and ASC-deficient mice demonstrated that Esx-1-dependent inflammasome activation exacerbated disease without restricting bacterial growth, indicating a host-detrimental role of this inflammatory pathway in mycobacterial infection. These findings define an immunoregulatory role for Esx-1 in a specific host-pathogen interaction *in vivo*, and indicate that the Esx-1 secretion system promotes disease and inflammation through its ability to activate the inflammasome.

## Author Summary

With ∼2 million people dying from tuberculosis every year, *Mycobacterium tuberculosis* represents the single most important bacterial pathogen globally. We use the closely related *Mycobacterium marinum* to study fundamental aspects of mycobacterial pathogenesis, likely to extend to human tuberculosis. The Esx-1 (type VII) secretion system is a major virulence determinant of pathogenic mycobacteria, including *M. tuberculosis* and *M. marinum*. However, a molecular explanation for Esx-1-mediated virulence *in vivo* has been lacking. Here we address this problem in a non-lethal mouse model of *M. marinum* infection that allows quantitative analysis of disease progression. *M. marinum* established local infection with important features of human tuberculosis, including formation of granulomas with caseating centers. Using a combination of bacterial and host mutants, we show that Esx-1-mediated activation of the host inflammasome increases inflammation without restricting bacterial growth, suggesting that activation of the inflammasome during mycobacterial infection is a manifestation of bacterial virulence rather than a manifestation of host response. These findings define a biological role for Esx-1 in a specific host-pathogen interaction *in vivo*, and imply that the Esx-1 secretion system has evolved specifically to promote host pathology.

## Introduction

One third of the world's population is infected with *Mycobacterium tuberculosis*, a human specific pathogen responsible for nearly 2 million deaths annually [1]. To facilitate fundamental studies of *M. tuberculosis* infection, safer and experimentally more amenable species are often used as models. Among these, the closely related *M. marinum* is used increasingly to study pathogenesis [2], [3]. *M. marinum* is a pathogen of fish and amphibians causing disease with many features of tuberculosis [2], and is also able to infect immunocompetent humans where it induces formation of dermal granulomas pathologically similar to those formed in tuberculosis [2], [4]. Importantly, the Esx-1 (Early secreted antigen 6 kilodaltons [Esat-6] secretion system 1) secretion system is highly conserved between *M. tuberculosis* and *M. marinum*, and required for virulence of both species [5]–[8]. Esx-1 is encoded primarily by genes within the chromosomal region of difference 1 (RD1) [9]; indeed, attenuation of the *Mycobacterium bovis* BCG vaccine strain is in large part due to a deletion of RD1, emphasizing the general significance of this secretory apparatus in mycobacterial virulence [10]. However, the biological function of Esx-1 during infection remains incompletely understood.

Macrophages infected with mycobacteria secrete proinflammatory cytokines, including IL-1β and IL-18 [11]–[15]. Both *M. tuberculosis* and *M. marinum* induce secretion of IL-1β in an Esx-1-dependent manner *in vitro* [13], [14]. The cysteine protease caspase-1 is a critical component of inflammasomes and is required for proteolytic activation and release of IL-1β and IL-18. Analysis of *M. marinum* infection demonstrates that Esx-1 is required to activate an inflammasome containing NLRP3 (Nalp3) and ASC [13]. In agreement with these findings, *M. bovis* BCG, which lacks Esx-1, is unable to activate caspase-1 efficiently [16]. However, nothing is known about the relevance of inflammasome activation to the progression of mycobacterial infection *in vivo*.

We examined the role of the inflammasome in *M. marinum* infection of mice by a ‘genetics squared’ approach, in which host and pathogen genetic strategies are combined in a single experimental system [17]. By this approach we are able to attribute a pathogenic role for Esx-1 in a defined host-pathogen interaction *in vivo*.

## Results

### Quantification of disease and inflammation demonstrates a requirement for Esx-1 in *M. marinum* virulence in mice

**Figure 1:**
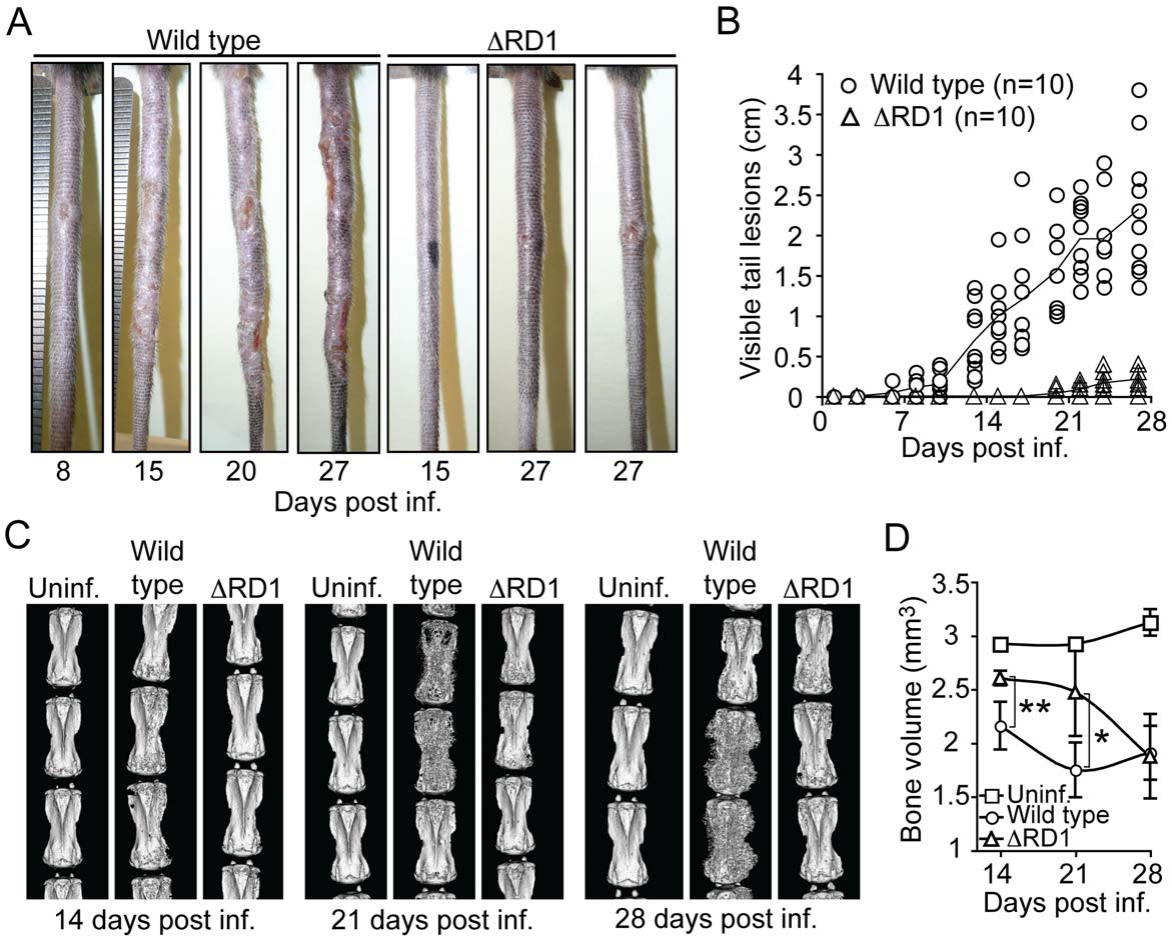
Disease and inflammation can be quantified and indicate a major role for Esx-1 in virulence. (**A**) B6 mice were infected via tail vein injection with either wild type or ΔRD1 bacteria, and observed for appearance of visible tail lesions. Shown are representative tails at indicated times post infection. (**B**) Quantification of the accumulated length of all visible lesions in individual tails at indicated times. Solid lines indicate the mean for each group. (**C**) Micro-CT scans of tails infected with wild type or ΔRD1 bacteria. Uninfected control mice were injected with PBS. Shown are 3D-renderings for one representative tail from each group at indicated times post infection. (**D**) Mean bone volume at indicated times. Values represent mean ± SD of three mice per group. Student's t-test (*P<0.05; **P<0.01).

Mice were infected via tail vein injection with wild type or Esx-1 deficient (ΔRD1) *M. marinum* and observed for development of disease (Figure 1). In wild type *M. marinum* infection, visible lesions appeared in tails ∼1 week post infection, and over time these lesions increased in size and became more numerous (Figure 1A). Determination of the accumulated length of all visible lesions in individual tails allowed quantitative kinetic analysis of disease progression (Figure 1B). Wild type *M. marinum* caused severe tail disease, whereas ΔRD1 infected mice developed very few, or no, lesions (Figure 1A, B). Moreover, lesions in ΔRD1 infected tails were small, and did not significantly increase in size over time. Complementation of ΔRD1 bacteria with the *M. tuberculosis*-derived RD1-locus (ΔRD1::RD1) restored ability to cause disease (Figure S1), confirming a specific role for Esx-1 in pathogenesis and, importantly, demonstrating functional conservation of this secretory pathway between *M. tuberculosis* and *M. marinum in vivo*.

Infected mice did not lose weight over the course of the experiments (Figure S2A), suggesting that *M. marinum* did not cause significant systemic effects. Tail infection was not simply the result of inoculation and growth at the site of injection because infection by intracardiac injection caused similar tail disease (Figure S2B), showing that *M. marinum* can spread via the blood to cause disease in this tissue. Thus, *M. marinum* cause disease confined to the tail, which is likely due the low optimal growth temperature (∼32°C) of the bacteria and the cooler environment provided in the tail [18].

Initial histopathological studies indicated significant bone erosion of vertebrae in tails of mice infected with wild type *M. marinum*, suggesting that direct and quantitative analysis of erosion could be used as a separate readout of inflammation caused by infection. To this end, we measured the bone volume of tail vertebrae over time by micro-computed tomography (micro-CT), a technique capable of quantifying inflammatory bone damage and subsequent repair in mice (Figure 1C, D) [19]. During the first 3 weeks of infection, wild type *M. marinum* induced considerably more bone loss than did ΔRD1, showing that wild type infection caused more bone erosion—and by inference, a stronger inflammatory response (Figure 1D). However, by 28 days post infection vertebrae from both ΔRD1 and wild type infections showed similarly reduced bone volume (Figure 1D), suggesting that infection with Esx-1-deficient bacteria does induce bone erosion, but with delayed kinetics. In addition, at this late time point, there was significant bone regeneration in tails of mice infected with wild type (Figure 1C; note massive sprouting of new bone in wild type infected tails at 28 days post infection), probably due to normal osteoblastic response to bone destruction [19] as well as to resolution of the acute phase of infection. Thus, two separate quantitative traits, visible lesions and bone volume, indicated that wild type *M. marinum* cause significantly more disease and inflammation than Esx-1-deficient bacteria.

### *M. marinum* cause formation of granulomas similar to those formed in tuberculosis

**Figure 2:**
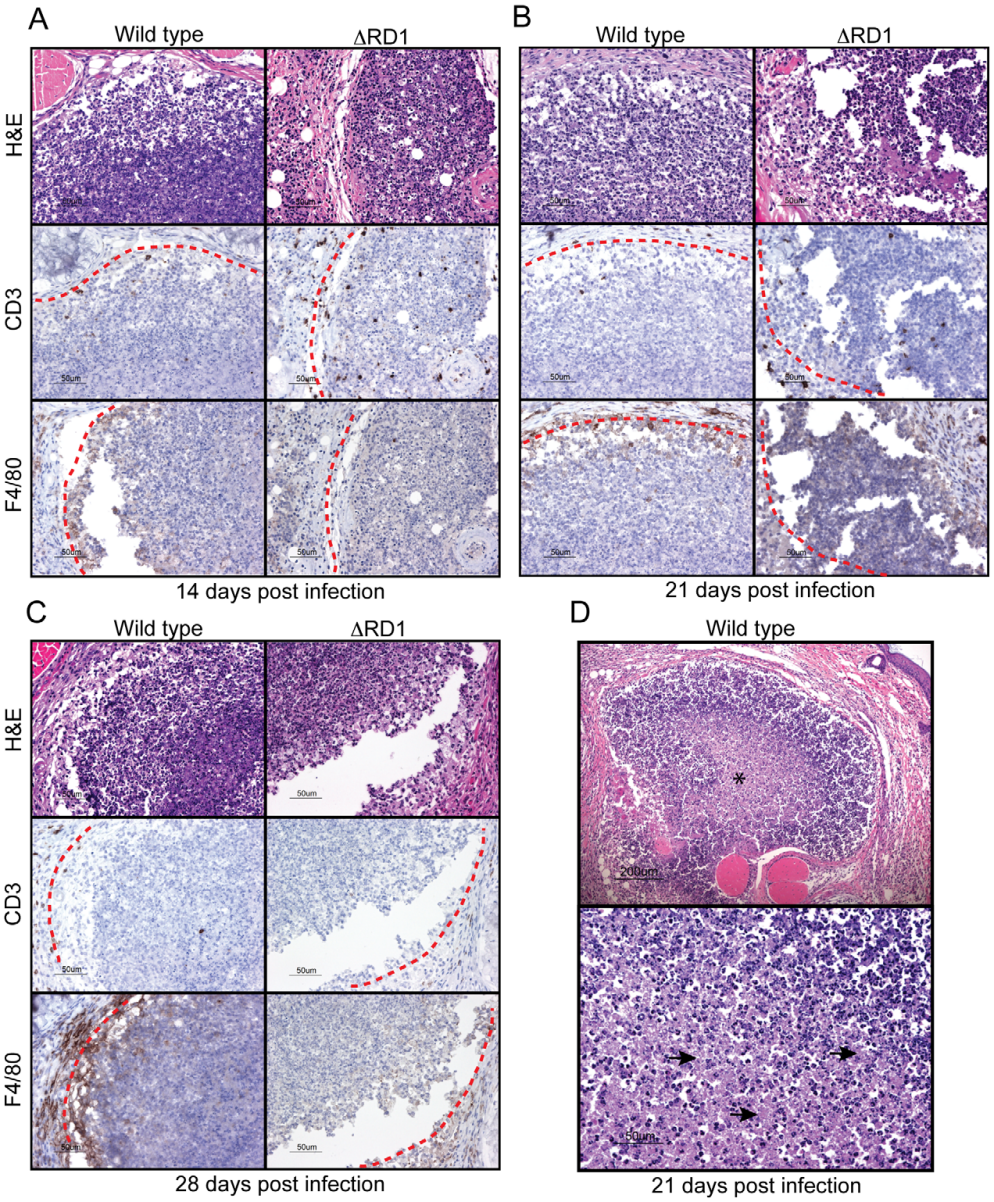
*M. marinum* cause formation of granulomas, similar to those formed in human tuberculosis, in an Esx-1-dependent manner. B6 mouse tails infected with wild type or ΔRD1 *M. marinum* were analyzed for formation of granulomatous lesions by hematoxylin and eosin (H&E), as well as anti-CD3 (T cells) and anti-F4/80 (macrophages) immuno histochemistry. Immunostained cells appear brown. Lesion borders are indicated with a red dotted line for clarity. (A) 14 days post infection. (B) 21 days post infection. (C) 28 days post infection. (D) Top panel: H&E staining of a typical granuloma in wild type infected tails 21 days post infection. Center contains acellular necrosis (indicated with an asterix). Lower panel: Higher magnification of region with acellular necrosis (representative areas are indicated with arrows).

Hematoxylin and eosin (H&E) staining showed PMN infiltration at sites of infection in both wild type and ΔRD1 infected tails one day post infection, and immunohistochemistry revealed few macrophages and T cells at this time (not shown). At 14 days post infection, lesions in wild type *M. marinum* infections demonstrated a peripheral ring containing macrophages and T cells, with granulomatous architecture (Figure 2A; Figure S3); the cellularity of this peripheral lining increased over time (Figure 2B, C; Figures S4 and S5), and developed into a solid border of macrophages and epitheloid macrophages with juxtaposed T cells by 21 days post infection. Granulomas in wild type infections exhibited central acellular necrosis from 14 days post infection, and the amount of central necrosis increased over time (Figure 2D; Figure S6). Thus, wild type *M. marinum* induced formation of granulomas with central caseous necrosis, histologically very similar to those formed in human tuberculosis, but distinct from those in murine infection with *M. tuberculosis*, which generally lack central necrosis. In contrast, the lesions present in infections by ΔRD1 *M. marinum* were smaller, did not develop into well-delineated granulomas during the timeframe of the infection (Figure 2A–C), and did not exhibit central necrosis until 28 days post infection (not shown), indicating that Esx-1 is required for a normal granulomatous response. During the first 21 days of infection ΔRD1 lesions also contained more T cells (Figure S7), which localized throughout the entire structure rather than organized to the periphery as in wild type infection (Figure 2A–C), implying that Esx-1 affects T cell functions *in vivo* via unappreciated mechanisms. After 28 days, however, few T cells were observed in both in wild type and ΔRD1 induced lesions (Figure S7; Figure 2C).

### *M. marinum* grows specifically in tails and escapes phagosomes in an Esx-1-dependent manner

**Figure 3:**
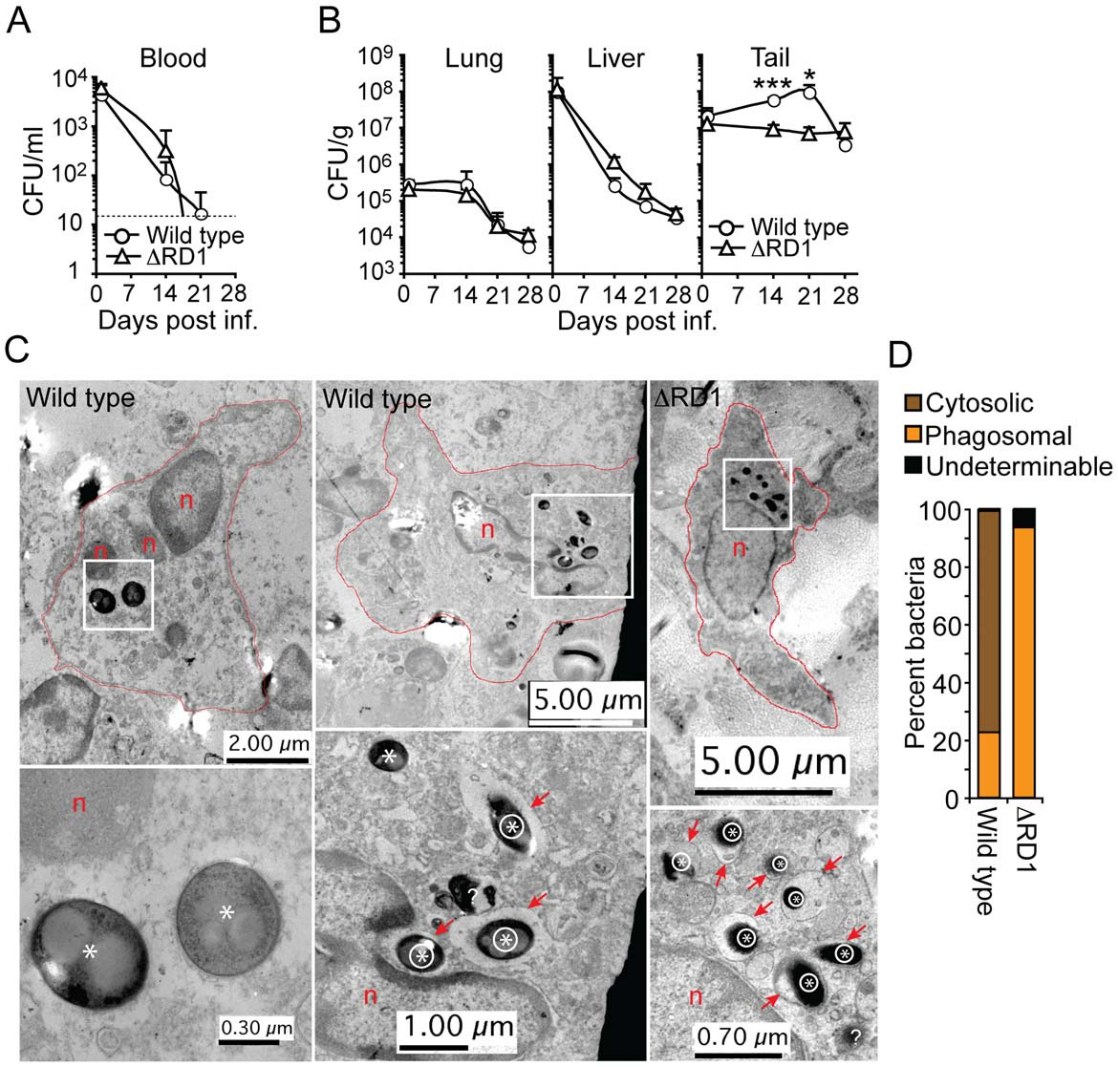
Esx-1 promotes bacterial growth and phagosome escape in tails of infected mice. (**A**) Bacterial burdens in the blood of wild type and ΔRD1 infected B6 mice at indicated time points. Dotted line indicates detection level. (**B**) Bacterial burden in lung, liver and tail tissues in wild type and ΔRD1 infected mice, as indicated. Values are mean + SD of three mice per group (A and B). The statistical difference between wild type and ΔRD1 infected tails was calculated with Student's t-test (*P<0.05; ***P<0.001). Similar statistical analysis indicated a significant (*P*<0.05) bacterial growth in wild type infected tails between day 1 and 21. The mean CFU/g tail tissue during the first 21 days in wild type infected mice were as follows: day 1: 1.8×10^7^; day 14: 5.82×10^7^; day 21: 9.95×10^7^. (**C**) TEM analysis of infected cells in lesions in wild type (left and middle panels) and ΔRD1 (right panel) infected tails. Upper panels: Cell morphology, degree of nuclear condensation, amount of cytoplasm and cytoplasmic granules suggested infected cells were macrophages. Cell membranes are indicated with red lines, nuclei with n, and areas with intracellular bacteria with white boxes. Lower panels: Higher magnification of areas with intracellular bacteria. Intraphagosomal bacteria are indicated with an encircled asterix, and cytosolic bacteria with an asterix. Red arrows point to membranes of bacteria-containing vesicles. Question mark indicates a bacterium with undeterminable localization. (**D**) Quantification of cytosolic and intraphagosomal wild type (n = 142) and ΔRD1 (n = 389) bacilli in infected tails by TEM. ‘Undeterminable’ indicates bacteria with uncertain localization.

To address the ability of wild type and ΔRD1 *M. marinum* to grow during infection, mice were analyzed for colony forming units (CFUs) in blood, lung, liver, and tail (Figure 3A, B). Similar numbers of wild type and ΔRD1 bacteria were retrieved from blood and the three tissues analyzed one day after infection. Subsequently, both strains were similarly cleared from blood, lung and liver, suggesting that *M. marinum* is seeded systemically upon injection, but is unable to colonize internal organs productively (Figure 3A, B). In contrast, both wild type and ΔRD1 bacteria maintained colonization in the tails, where wild type showed modest growth (Figure 3B). The number of wild type bacteria in infected tails dropped to the level of ΔRD1 between 21 and 28 days post infection, a feature that might be explained by the onset of an adaptive immune response, which is typically initiated ∼20 days post infection in *M. tuberculosis* infected mice [20], [21].

Analysis ∼2.5 and 4 months after infection demonstrated similar bacterial numbers (∼1×10^6^ CFU/g and ∼3×10^5^ CFU/g, respectively) in the tails of wild type and ΔRD1 infected mice (Figure S8A, B). Concomitant analysis of visible tail lesions demonstrated that disease induced by wild type *M. marinum* decreased to a level comparable to that of ΔRD1 over time (Figure S8C), suggesting that both strains are able to similarly persist in the tails with minimal pathology for extended periods of time. Thus, Esx-1 may exert its major pathogenic role during the acute phase of infection.

Histochemical analysis suggested that both wild type and ΔRD1 bacilli were scattered throughout the lesions in infected tails, with a preference for peripheral regions (not shown). Analysis of this region by transmission electron microscopy (TEM) in both wild type *M. marinum* and ΔRD1 lesions 21 days post infection indicated that bacteria resided preferentially in host cells with morphology consistent with macrophages (Figure 3C). Furthermore, 76.8% of wild type bacteria were observed without an apparent surrounding host membrane, whereas 93.6% of ΔRD1 bacteria were found within membranous vesicles (Figure 3C, D; Figure S9), suggesting that intracellular *M. marinum* escapes from phagosomes in an Esx-1-dependent manner *in vivo*.

### Esx-1 promotes secretion of proinflammatory IL-1β *in vivo*

**Figure 4:**
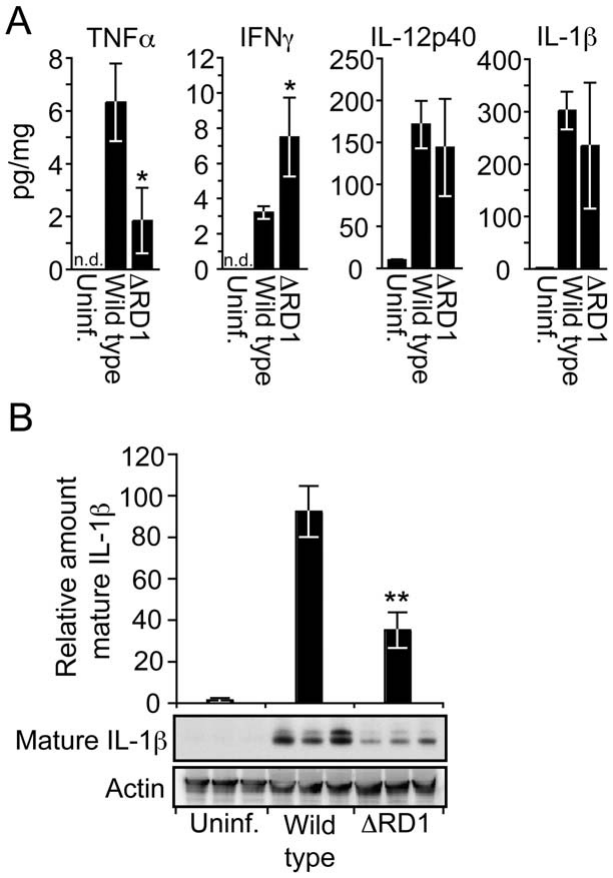
Esx-1 promotes secretion of IL-1β *in vivo*. (**A**) 20 days post infection tail suspensions were prepared from uninfected controls, wild type and ΔRD1 infected mice, and analyzed by Luminex for indicated cytokines. Data are presented as the amount of cytokine detected (pg/ml) divided by the total protein content of the suspensions (mg/ml). Values are mean ± SD of three mice per group. (**B**) Tail suspensions from three mice per group were separated by SDS-PAGE, and analyzed for mature IL-1β and actin by immunoblot. For each tail, the amount of mature IL-1β was divided by the amount of actin, and normalized to the wild type infected mouse with the highest ratio. Values are mean ± SD for each group. Student's t-test (*P<0.05; **P<0.01).

Tail specimens allowed for detailed analysis of proteins in the diseased tissue (Figure 4). Wild type *M. marinum* induced more TNFα and less IFNγ as compared to ΔRD1 (Figure 4A), suggesting a more proinflammatory response during wild type *M. marinum* infection. The amount of IL-12p40 was high but unaffected by Esx-1 (Figure 4A). Similarly, total IL-1β protein also was greatly increased in tails infected with both wild type and Esx-1-deficient bacteria (Figure 4A).

IL-1β is synthesized as a ∼31 kDa inactive proprotein, which is secreted to the extracellular environment after proteolytic processing into its biologically active mature form (∼17 kDa) by caspase-1. To examine the amount of mature IL-1β specifically, we analyzed tail proteins by Western blot, which separates mature from pro-IL-1β by molecular weight. Such analysis demonstrated a 2.6-fold increase of mature IL-1β in the tails of mice infected with wild type *M. marinum* compared to ΔRD1 infection (Figure 4B), suggesting that Esx-1 promotes caspase-1 activation *in vivo*. Because IL-1β has significant pro-inflammatory effects, this feature may contribute to the dramatic difference in inflammation between wild type and ΔRD1 infections.

### Esx-1 is required for *M. marinum* activation of the NLRP3/ASC-inflammasome *in vitro*

**Figure 5:**
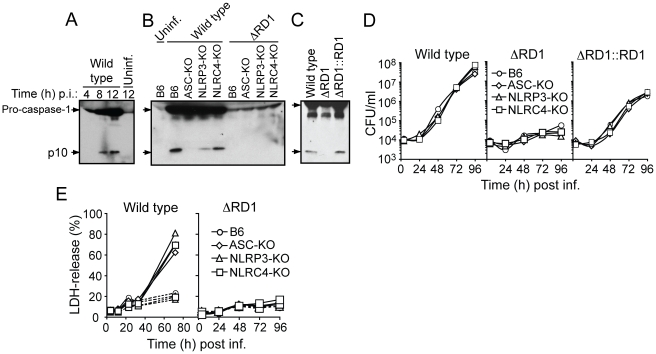
Esx-1 is required for activation of the NLRP3/ASC-inflammasome in bone marrow-derived macrophages. (**A**) B6 macrophages were infected with wild type *M. marinum* for 4, 8 and 12 hrs, as indicated, and analyzed for caspase-1 activation by anti-caspase-1 p10 immunoblot. Uninfected macrophages were analyzed as control. (**B**) Macrophages were infected as indicated, and analyzed for caspase-1 p10 12 hrs post infection. (**C**) B6 macrophages were infected as indicated, and analyzed for caspase-1 p10 12 hrs post infection. (**D**) Macrophages were infected (MOI = 0.1) as indicated, and bacterial growth determined by CFU-analysis. (**E**) Macrophages were infected with wild type (left panel) or ΔRD1 (right panel) and analyzed for cell death. Solid lines represents infected cells, and dotted lines represents uninfected controls. At indicated time points, supernatants were analyzed for LDH-release as a measure of loss of host cell membrane integrity (i.e. host cell death). Data are presented as relative LDH-release; 100% LDH-release was defined by lysis of uninfected cells with Triton-X100 treatment. Shown are representative data for at least three separate experiments (A to E).

Caspase-1 is autoprocessed into 20 kDa (p20) and 10 kDa (p10) subunits upon assembly of an inflammasome. These subunits become part of the active inflammasome, and can also be used as markers for caspase-1 activation in Western blot analysis. Kinetic analysis in B6 macrophages infected with wild type *M. marinum* showed that caspase-1 p10 appeared 8 hrs post infection, suggesting that the bacteria interacted with the host cytoplasm at this time to activate an inflammasome (Figure 5A). Detailed analysis of bacterial and host genetic requirements demonstrated that wild type but not ΔRD1 activated caspase-1 in a process involving the host proteins ASC and NLRP3 but not NLRC4 (Ipaf), indicating that *M. marinum* activates the NLRP3/ASC-inflammasome in an Esx-1-dependent manner (Figure 5B). Of note, infection with wild type *M. marinum* induced higher levels of pro-caspase-1 than ΔRD1, independent of the inflammasome (Figure 5B), which might be explained by secretion of Esat-6, a major Esx-1 substrate that has been proposed to induce caspase-1 gene expression in macrophages [22]. Like wild type, ΔRD1::RD1 bacteria caused caspase-1 activation in infected macrophages, indicating a specific role for Esx-1 in this process and further emphasizing the functional conservation of Esx-1 between *M. tuberculosis* and *M. marinum* (Figure 5C). In agreement with these findings, and with a previous analysis of cytokine secretion from mycobacteria infected macrophages [13], Esx-1-proficient bacteria induced ASC- and NLRP3-dependent secretion of IL-1β and IL-18 (Figure S10).

Analysis of TNFα and IL-6 demonstrated Esx-1-dependent secretion (Figure S11), implying that Esx-1 promotes NFκB activation in macrophages *in vitro*, which could account for the increased TNFα seen in infections by wild type *M. marinum in vivo*. However, in agreement with previous reports [5], [23], IL-12p40 secretion was repressed in macrophages infected with wild type *M. marinum* (Figure S11), implying that Esx-1 down-regulates secretion of this cytokine via NFκB-independent mechanisms. The finding that wild type and ΔRD1 *M. marinum* caused similar levels of IL-12p40 in infected tails (Figure 4A) might be explained by additional stimulation provided in the complex *in vivo* environment, or by contributions from other cell types [24].

Activation of inflammasomes is normally accompanied by pyroptosis, whereby infected host cells succumb to a necrotic-like cell death commonly believed to represent an altruistic mechanism to restrict bacterial growth [25]. As expected, wild type but not Esx-1-deficient bacteria were able to grow in infected macrophages (Figure 5D). However, bacterial growth was unaffected by the inflammasome, as wild type *M. marinum* grew similarly well in B6, ASC-KO and NLRP3-KO macrophages (Figure 5D). Moreover, while *M. marinum* caused cytotoxicity to infected macrophages in an Esx-1-dependent manner, this feature was similarly independent of the inflammasome (Figure 5E). Thus, *M. marinum*-induced activation of the inflammasome is separable from its effect on macrophage viability, and intracellular bacterial growth is unaffected by inflammasome activation *in vitro*.

### Esx-1-dependent activation of the inflammasome exacerbates inflammation without restricting bacterial growth *in vivo*

**Figure 6:**
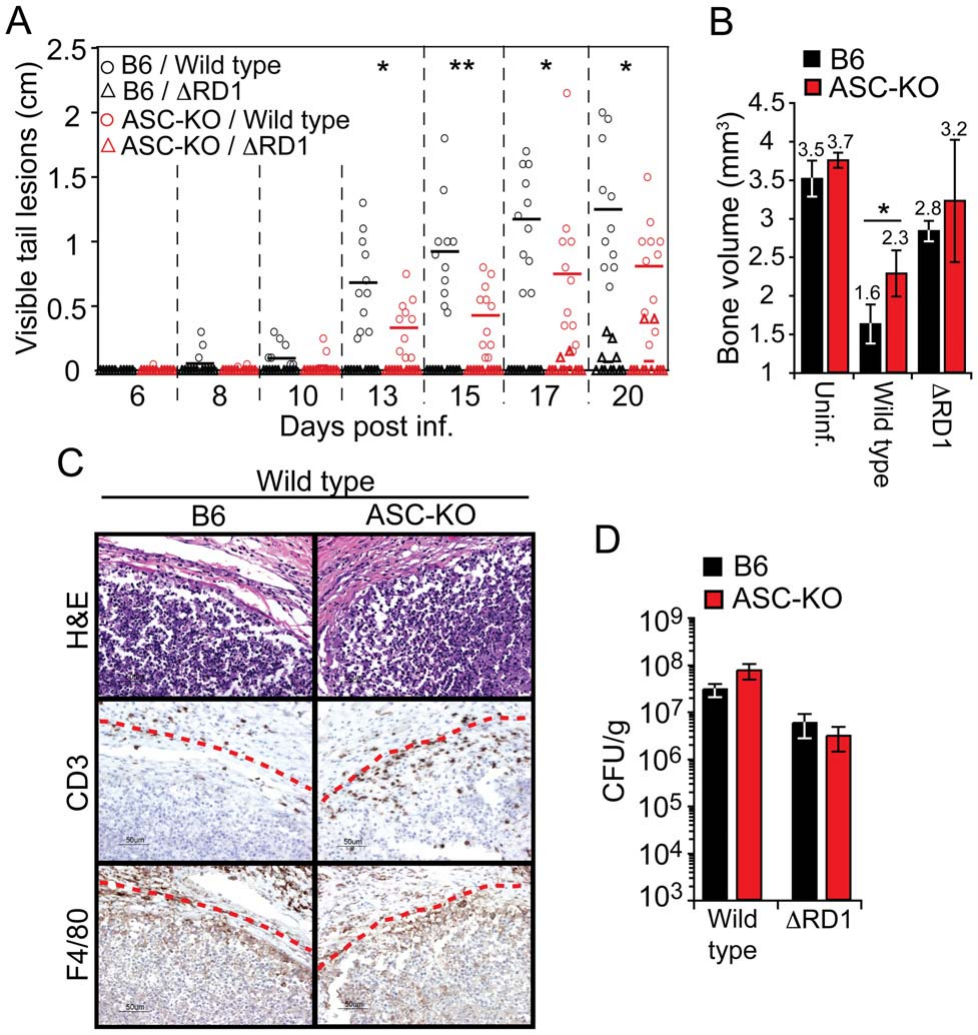
Activation of the inflammasome exacerbates inflammation without restricting bacterial growth *in vivo*. (**A**) Mice were infected as indicated, and analyzed for accumulated length of all visible tail lesions. Lines indicate the mean for each group (n = 11 per group). (**B**) Mean ± SD bone volume determined by micro-CT of three mouse tails for each group at 21 days post infection. Statistical significance of differences between B6 and ASC-KO mice infected with wild type *M. marinum* was calculated by Student's t-test (*P<0.05; **P<0.01) (A and B). (**C**) 21 days post infection; tails of B6 and ASC-KO mice were analyzed for granuloma formation by H&E as well as anti-CD3 and anti-F4/80 immunohistochemistry. Lesion borders are indicated with a red dotted line for clarity. (**D**) Bacterial burdens in tails of infected mice, as indicated, at 21 days post infection. Values are mean CFU ± SD of three mice per group.

Because these *in vitro* experiments left uncertain how inflammasome activation affected the course of mycobacterial infection *in vivo*, B6 and ASC-KO mice were infected with wild type and ΔRD1 bacteria, respectively (Figure 6). Analysis of visible tail lesions and bone volume of tail vertebrae showed that development of disease was dependent on Esx-1 in both mouse strains (Figure 6A, B). However, infection with wild type *M. marinum* caused less visible pathology in ASC-KO than in B6 mice (Figure 6A), indicating that deficient inflammasome activation results in less disease. Consistent with this finding, micro-CT-analysis 21 days post infection demonstrated reduced loss of bone volume in tail vertebrae in ASC-KO compared to B6 mice upon challenge with wild type *M. marinum* (Figure 6B), confirming that lack of inflammasome activation leads to a milder inflammatory response. Thus, Esx-1-dependent activation of the inflammasome causes increased disease and inflammation in infected mice.

Histological analysis of granulomas formed in tails of B6 and ASC-KO mice infected with wild type *M. marinum* was performed 21 days post infection, and demonstrated similar overall architecture and cellularity of granulomas formed in the two mouse strains (Figure 6C). Notably, granulomas in wild type *M. marinum* infections in both mouse strains showed caseous necrosis, consistent with the ASC-independence of *M. marinum*-induced macrophage death *in vitro*. However, granulomas in ASC-KO mice contained increased numbers of T cells as compared to B6 mice (Figure 6C), suggesting that Esx-1's effect on T cells (Figure 2; Figure S7) may in part be mediated via the inflammasome.

The decreased inflammation observed in ASC-KO mice cannot be explained by decreased bacterial growth, because in agreement with our *in vitro* findings (Figure 5D), CFU analysis indicated a similar bacterial burden in tails of wild type infected ASC-KO and B6 mice (Figure 6D). Taken together, these findings demonstrate that Esx-1-dependent activation of the inflammasome *in vivo* exacerbates disease and inflammation without significantly limiting bacterial growth, suggesting that inflammasome activation is detrimental to the host in mycobacterial infection.

## Discussion

Experimental infections of laboratory animals are increasingly important to our understanding of microbial pathogenesis, as these may elucidate mechanisms by which pathogens exploit the host that might not be appreciated using reductionist *in vitro* models. In addition, the ability to manipulate both host and pathogen genetically has become increasingly important for understanding the molecular basis of virulence [17]. The importance of this ‘genetic squared’ approach is well illustrated by the study of *M. marinum* infection, in which host-pathogen interactions have been dissected extensively *in vitro* and *in vivo* using infections of both Drosophila and zebrafish embryos [26]–[29]. However, the fruit fly and zebrafish embryos lack aspects of a mammalian immune system, including functional T cells, which are generally believed to play an important role in the host response to mycobacteria. Interestingly, early work in the 1960s through 1980s demonstrated that *M. marinum* is able to infect primarily cooler anatomical regions in mice [18], [30]. However, neither mycobacterial genetics nor mouse immunology was sufficiently developed to take full advantage of this model, and it has largely been abandoned for the past 30 years. In light of recent advances in these areas, we re-examined this model and found that its unique features allow a detailed analysis of infection leading to new insights into the biological role of Esx-1, a major virulence determinant generally involved in mycobacterial pathogenesis [2], [9], [31].

*M. marinum* injection into mice caused local infection in the tail. Measurement of disease by two separate analyses, the visible area of diseased tissue and the extent of bone destruction, demonstrated a major role in pathogenesis for the Esx-1 secretion system. The difference in pathology is likely not due to the difference in bacterial growth between the two strains, because this difference was small and resolved completely by 28 days, a time point at which the difference in disease was still significant. Furthermore, after the initial acute phase of infection (≥28 days), both wild type and ΔRD1 bacilli persisted equally well, with little pathology in tails of infected animals, suggesting Esx-1-independent establishment of latent disease. Taken together, these findings suggest that a major role for Esx-1 *in vivo* is to manipulate the inflammatory response during the early events of infection. This is consistent with the recent discovery of an important role for Esx-1 in *M. marinum* infected zebrafish embryos, which, unlike adult zebrafish, lack a functional adaptive immune response [32]. That study found a role for Esx-1 in promoting bacterial spread and expansion of granulomatous lesions, in part by influencing macrophage chemotaxis [32].

Because Esx-1-dependent pathology appeared to relate to perturbation of the host immune response, we analyzed cytokines in tails infected with wild type and Esx-1-deficient bacteria. Quantification of TNFα and IFNγ suggested that Esx-1 alters the immune response by decreasing the T cell response and enhancing inflammatory cytokine production. IL-12p40 was unaffected by Esx-1 *in vivo*, which differs from the markedly decreased levels of IL-12p40 secretion observed in wild type infected macrophages *in vitro*. This apparent paradox might be explained by *in vivo* contribution from dendritic cells, whose secretion of IL-12p40 is not repressed by infection with Esx-1-proficient *M. tuberculosis* [11]. Previous analyses have suggested that secretion of TNFα is unaffected or even decreased during macrophage infection with wild type compared to Esx-1-deficient mycobacteria [5], [13]. In contrast, our findings indicated that secretion of this cytokine is enhanced by infection with Esx-1-proficient bacteria both *in vivo* and in non-primed macrophages *in vitro*. While the reasons for these differences remain unknown, these anomalies stress the importance of translating *in vitro* findings into the more complex *in vivo* environment.

IL-1β is upregulated in the lungs of tuberculosis patients [33], and both IL-1β and IL-18 are secreted from *M. tuberculosis* and *M. marinum* infected macrophages *in vitro* [11], [13]. While IL-18 might have a minor role in experimental *M. tuberculosis* mouse infections [34], [35], IL-1β is commonly believed to play a role in the host response elicited by mycobacteria. IL-1β was highly upregulated in *M. marinum* infected mouse tails, and Esx-1 promoted processing of this cytokine into its biologically active form *in vivo*. Studies in primary macrophages indicated that *M. marinum* activates the NLRP3/ASC-inflammasome in an Esx-1-dependent manner. However, while *M. marinum* caused Esx-1-dependent cell death to infected macrophages, this was not dependent on ASC or NLRP3, suggesting that the macrophage death so apparent *in vitro* and possibly underlying caseous necrosis *in vivo* is distinct from caspase-1 activation. Indeed, ASC was dispensable also for development of caseation *in vivo*.

A role for inflammasomes in determining the fate of cellular macrophage infections has been analyzed *in vitro* for several bacterial pathogens [25], [36]. However, the biological role of inflammasomes *in vivo* remains elusive. For *M. marinum*, activation of the inflammasome exacerbated disease and inflammation without significantly limiting bacterial growth, indicating that inflammasome activation is detrimental to the host in mycobacterial infection and that disease, at least in part, is a function of the inflammatory response rather than direct bacterial mechanisms. Possibly Esx-1 has evolved to increase the inflammatory response in order to promote bacterial spread to new hosts, as when granulomas rupture into bronchi during tuberculosis, or into the skin during piscine infection by *M. marinum*. Interestingly, however, ASC-deficiency does not completely abolish the ability of wild type bacteria to cause disease and inflammation, suggesting that inflammasome activation is part of a broader repertoire of Esx-1-mediated virulence mechanisms; it is likely that Esx-1-mediated regulation of TNFα and IFNγ, as well as Esx-1-mediated activation of the host metalloprotease MMP-9 [37], also contributes to inflammation and disease progression.

In infected host cells *in vitro*, a fraction of *M. marinum* bacilli escapes the phagosome in an Esx-1-dependent manner [8], [38], which may promote bacterial spread to uninfected neighboring cells [8], [38]. While the ability of *M. tuberculosis* to escape the phagosome remains highly controversial, Esx-1-dependent communication with host cell cytoplasm might play a similar role also in *M. tuberculosis* infection, and might also contribute to MHC class I presentation of mycobacterial antigens [6], [39]–[42]. Our study suggests that phagosome escape occurs *in vivo* as well as *in vitro*, a point previously uninvestigated. Thus, this may be a pathogenic role for the Esx-1 secretion system during infection, and might explain the requirement for Esx-1 in activation of the inflammasome, which generally responds to cytoplasmic signals.

Infection with wild type *M. marinum* caused formation of granulomas with a cellularity and architecture similar to those formed in tuberculosis [43], [44]. The granulomas also developed central caseating necrosis, an important feature in *M. marinum* infected fish and in human tuberculosis not replicated in the *M. tuberculosis* mouse model [7], [43], [45]. Thus, the mouse model of *M. marinum* infection might provide unique opportunities to study the development of caseous necrosis in the context of an experimentally amenable mammalian immune system. In contrast, Esx-1-deficient bacteria were unable to attract significant numbers of macrophages or to induce formation of proper granulomas, implying that Esx-1 has evolved to actively influence the genesis of granulomas. A similar requirement for Esx-1 has been observed in zebrafish embryos, where Esx-1-deficient bacteria are able to grow within macrophages but unable to recruit new macrophages to sites of *M. marinum* infection and induce their aggregation into granulomatous structures [7]. Importantly, our analysis extends this work and suggests that Esx-1's role in granuloma formation is significantly more complex, since compared to wild type, lesions formed in mouse tails in response to ΔRD1 *M. marinum* exhibited earlier T cell recruitment, aberrant T cell distribution, and a significant delay in developing central necrosis. Intriguingly, granulomas formed in response to wild type *M. marinum* in ASC-KO mice also exhibited increased numbers of T cells compared to similarly infected B6 mice, implying that at least part of Esx-1's effect on T cells is mediated via the inflammasome; this might be explained by IL-1β, which has been shown to functionally impair antigen presenting dendritic cells [46]. Although this hypothesis is consistent with data indicating that virulent *M. tuberculosis* decreases T cell activation by dendritic cells *in vivo* [47], future studies will be required to elucidate a possible role for Esx-1 and the inflammasome in influencing the adaptive host response during mycobacterial infection.

Our findings in the *M. marinum*-mouse model confirm and extend knowledge gained from other more established model systems, including the *M. marinum*-zebrafish and *M. tuberculosis*-mouse models. One advantage of the model we describe over zebrafish infection is the greater ability to probe the host immune response, particularly adaptive immunity. At the same time, this model is infection in a non-natural host, a defect that is shared by *M. tuberculosis* infections of rabbits, guinea pigs, and mice. As a result, there are almost certainly important adaptations *M. marinum* has made to its piscine and amphibian hosts that will not be discovered in murine infection, and it is equally likely that many adaptations *M. tuberculosis* has made to its human host will not be reflected in our model. Future studies are needed to establish how closely the *M. marinum*-mouse model mimics events in human tuberculosis. For example, it remains to be confirmed that granuloma formation in the tail progresses through the same mechanisms as in the lung of tuberculosis patients; initial insight into this important question may come from experimental aerosol infection of guinea pig or rabbit with wild type and Esx-1-deficient *M. tuberculosis*.

In summary, the mouse model of *M. marinum* infection has unique features that open up new avenues to analyze fundamental aspects of mycobacterial pathogenesis. Here we demonstrate that Esx-1-dependent activation of the inflammasome is host-detrimental, identifying an immunoregulatory function for Esx-1 in a defined host-pathogen interaction *in vivo* and suggesting that activation of caspase-1 during mycobacterial infection is a manifestation of bacterial virulence rather than a manifestation of host response.

## Methods

### Ethics statement

All animal studies followed the ethical guidelines of the *M. marinum* mouse infection protocol and the mouse bone marrow-derived macrophage protocol, which were created by FC, CD, JK and EJB and received ethical approvals by the Institutional Animal Care and Use Committee (IACUC) at Genentech.

### Bacterial strains

Wild type *M. marinum* M-strain and an isogenic deletion mutant lacking RD1 (ΔRD1) have been described previously [48]. ΔRD was complemented with RD1-2F9 by integration of this cosmid into the chromosomal attB-site [13].

### Macrophage infections

Bone marrow derived macrophages (BMDM) were obtained and cultured from C57BL/6 wild type, ASC-KO, NLRP3-KO and NLRC4-KO mice as described previously [13]. For analysis of caspase-1 activation and cytokine secretion, 3×10^6^ BMDMs/well were infected at an MOI of 5, essentially as described [13]. For analysis of bacterial intracellular growth and LDH-release, 5x10^4^ BMDMs/well were infected at an MOI of 5 or 0.1, as indicated in figure legends. All infections were performed at 32°C.

For analysis of caspase-1 activation and cytokine secretion upon *M. marinum* infection of bone marrow-derived macrophages, supernatants from infected cells were collected at indicated time points and immediately supplemented with complete, EDTA-free, protease inhibitor cocktail (Roche). Suspensions were centrifuged (5.500 rpm, 10 min, 4°C) to pellet remaining bacteria and cells, and subsequently concentrated 3-fold using Vivaspin 15R (2.000 MWCO; Sartorius Biolab). For Western blot analysis of caspase-1 activation, equal amounts were separated by SDS-PAGE. Caspase-1 p10 was detected with polyclonal rabbit anti-mouse caspase-1 p10 (M-20) Abs (Santa Cruz Biotechnology) followed by donkey anti-rabbit HRP-conjugated secondary Abs, and membranes were developed with West Pico (Pierce). Cytokines were measured by Luminex analysis (see below). For analysis of intracellular bacterial growth, infected macrophages were lysed with 0.1% (final concentration) Triton-X for 10 min at indicated time points, and serial dilutions were plated on 7H10 plates for CFU analysis. Cytotoxicity was assessed by analysis of LDH-release using cytotox 96 non-radioactive cytotoxicity assay (Promega), as described by the manufacturer. As control, uninfected macrophages were lysed with Triton-X, which causes complete lysis, as described above.

### Mouse infections

Female C57BL/6 (B6) mice and ASC-KO mice were infected with 1x10^7^ bacteria in 200 µl phosphate buffered saline (PBS) via tail vein or intracardiac injection, as indicated, at 12 weeks of age. Matched control mice were similarly injected with PBS. All ASC-KO mice used were backcrossed to B6 ≥18 times.

For mouse infections, bacteria were grown to logarithmic growth phase (OD_600_ = 0.7±0.2) in 7H9-broth, and collected by centrifugation (3500 rpm, 10 min). Cells were washed twice in PBS, and needled three times through a 26G1/2 needle (Becton Dickinson) to disrupt bacterial aggregates. Aggregates were pelleted by two separate centrifugation steps (2000 rpm, 1 min), where the supernatants, enriched for single cell bacteria, were transferred to new tubes. Bacterial suspensions were subsequently analyzed by light microscopy to confirm the absence of aggregates. Finally, the bacterial concentration was determined using a hemacytometer, and suspensions were diluted to 5×10^7^ bacteria/ml (final concentration).

### Analysis of visible tail lesions and bone erosion

The length (broadest width) of individual visible lesions was measured at indicated time points, and the accumulated length of all lesions in individual tails was calculated, and presented in centimeters.

Micro-computed tomography (micro-CT) imaging was performed on an *ex-vivo* micro-CT scanner (microCT 40, SCANCO Medical, Switzerland) at 12 µm isotropic voxel size, 1000 projections/rotation, 300 ms integration time, 70 keV photon energy, and 114 µA current. For each mouse, three corresponding tail vertebrae at or near the site of infection were scanned (except for the 14 days post infection time point in Fig 1C and D, where two vertebrae per mouse were scanned). The bone was segmented by applying a lower threshold (0.738 gHA/cc) to the 3D image data sets. Mean bone volume within the segmented bone was measured for each vertebra, and the average bone volume was calculated for each animal. Image analysis was performed using Analyze software package (AnalyzeDirect, Inc., Lenexa, KS, USA).

### Analysis of bacterial growth *in vivo*

Blood collected via cardiac puncture was subjected to serial dilutions and plated on 7H10 plates and the amount of bacteria presented as CFUs per ml. Tails were cut into ∼5 mm pieces and homogenized in 3 ml DMEM supplemented with 0.1% Triton-X, using an AHS200 homogenizer (VWR) with saw tooth adaptors (10×105 mm, Troemner). Livers and lungs were similarly homogenized. Organ and tail suspensions were serially diluted and plated on 7H10 plates. Bacterial load is presented as CFUs/gram tissue.

### Preparation of tail suspensions and cytokine analysis

Tails were severed from mice at the tail base, immediately bagged and put on dry ice, frozen in liquid N_2_ and pulverized with a similarly chilled biopulverizer (Biospec Products Inc.). Pulverized tails were resuspended in 1 ml PBS supplemented with complete, EDTA-free, protease inhibitor cocktail, and left on ice for 1.5 h. Finally the suspensions were centrifuged (20.000g, 20 min, 4°C) twice to pellet debris, and supernatants were collected for analysis. Total protein content in each tail suspension was determined by Bradford analysis (Bio-Rad). The amount of indicated cytokines was measured by Luminex analysis (see below), and for each tail, the amount of cytokine detected (pg/ml) was normalized to the total amount of protein (mg/ml) in that tail suspension, as determined by Bradford analysis. For Western blot analysis of mature IL-1β, similar amounts of samples were separated by SDS-PAGE. Mature IL-1β was detected with purified hamster anti-mouse IL-1β Abs (1 µg/ml final concentration, BD Biosciences Pharmingen) followed by goat anti-hamster HRP-conjugated secondary Abs. As loading control, actin was analyzed with affinity purified rabbit anti-actin Abs (Sigma) followed by donkey anti-rabbit HRP-conjugated Abs. Membranes were developed using a ChemiDoc XRS system (Bio-Rad) and the relative amounts of mature IL-1β and actin were quantified using Quantity One software (Bio-Rad). For each tail analyzed, the amount of mature IL-1β was divided by the amount of actin detected, and all values were subsequently normalized to tail with the highest ratio (i.e. most mature IL-1β).

### Luminex analysis

The concentration of indicated cytokines was determined using the Luminex 100 system (Luminex Corporation) run by the Bio-Plex Manager 5.0 software (Bio-Rad). All cytokines were measured using Bio-Plex reagent kits (Bio-Rad), and curve fitting was performed either by a Logistic-5 PL or 4-PL regression method.

### Histological analysis of granulomatous lesions

Tails were fixed in 10% buffered formalin followed by decalcification in Immunocal (Decal Chemical Corp) for 48 hours. Five transverse 3 µm sections, which included soft tissue and coccygeal vertebrae, were evaluated for each animal (at least 2 animals were analyzed per group). Sections of tails were stained with hematoxylin and eosin (H&E) for routine histologic evaluation, or for immunohistochemical evaluation, with either rat anti-mouse F4/80 (Serotec, Raleigh NC) at 10 ug/ml or with rabbit anti-CD3 clone SP7 (Lab Vision, Fremont CA) at a dilution of 1∶200. Photomicrographs were captured using a Nikon DXM1200C digital camera and images shown are at either 10× or 40× magnification. We scored a granuloma as caseating if there was acellular, amorphous eosinophilic material centrally located in an inflammatory lesion. For quantification of CD3-positive cells, images were acquired using the Ariol SL-50 automated slide scanning platform (Genetix Ltd, Hampshire, UK) at 100× final magnification. Using these scans, lesions from wild type and ΔRD1 infected tails were selected and defined by a pathologist in a blinded manner. CD3-positive cells within the defined lesion areas were identified and counted using Ariol's proprietary cell counting algorithm.

### Transmission electron microscope (TEM) analysis of granulomatous lesions

Cross sections (∼1 mm thickness) of formalin fixed tails were cut out. Sections were washed three times in 0.1 M sodium cacodylate buffer containing 3 µM calcium chloride for 15 min each, and then incubated with 1% osmium tetroxide, 0.8% potassium ferrocyanide, 3 µM calcium chloride in 0.1 M sodium cacodylate for 1 hour. After washing with distilled water three times for 15 min each, samples were stained and stabilized in ice-cold 2% uranyl acetate for 1 hour and dehydrated in an ethanol series of 20%, 50%, 70%, 90% and three times 100% successively for 3 min each. After washed with propylene oxide (EMS) two times for 3 min each, the samples were then infiltrated in well-mixed 50% propylene oxide, 50% Epon-812 (EMS) two times for 4 hours with agitation followed by 100% Epon-812 three times for 4 hours each with agitation, after which the samples were placed in an oven and allowed to polymerize at 60–80°C for 48 hours. Thick section (∼1 µm) were performed and stained with 1% toluidine blue for the selection of granulomas. The selected areas were trimmed for thin section. Thin sections (∼80 nm) were collected and pre-stained with 2% uranyl acetate and lead citrate before examination in an FEI CM12 TEM.

## Supporting Information

Figure S1 Complementation of *M. marinum* ΔRD1 bacteria with the *M. tuberculosis*-derived RD1-locus restores ability to cause disease. B6 mice were infected with 1×10^7^ wild type, ΔRD1 or ΔRD1::RD1 bacteria via tail vein injection, as indicated. (A) Shown is representative tails 15 days post infection. (B) Quantification of the accumulated length (in cm) of all visible lesions in individual tails of wild type, ΔRD1 and ΔRD1::RD1 infected mice at 15 days post infection. Values represent mean of 10 mice per group. Statistical significance was calculated by the Student's t-test (* *P*<0.05, **P<0.01, ***P<0.001).
(3.61 MB TIF)

Figure S2 *M. marinum* cause local disease in the tail. (A) B6 mice were infected with 1×10^7^ bacteria via tail vein injection as indicated, and monitored for weight changes. Weight development was unaffected by infection, suggesting that *M. marinum* does not cause significant systemic effects. Control mice were similarly injected with PBS. (B) B6 mice were infected with 1×10^7^ wild type *M. marinum* via intra cardiac injection and followed over time for appearance of lesions. Lesions (indicated with red arrow) were observed in the tail of infected animals ∼15 days post infection, suggesting that the bacteria spread via the blood and specifically established an infection in the tail.
(3.01 MB TIF)

Figure S3 Histological analysis of granulomas 14 days post infection. High magnification of data presented in Figure 2A. For clarity, examples of immunostained cells are indicated with red arrowheads.
(9.43 MB TIF)

Figure S4 Histological analysis of granulomas 21 days post infection. High magnification of data presented in Figure 2B. For clarity, examples of immunostained cells are indicated with red arrowheads.
(8.85 MB TIF)

Figure S5 Histological analysis of granulomas 28 days post infection. High magnification of data presented in Figure 2C. For clarity, examples of immunostained cells are indicated with red arrowheads.
(9.19 MB TIF)

Figure S6 Caseating centers in *M. marinum* wild type induced granulomas. High magnification of data presented in Figure 2D. Upper panel: H&E staining of a granuloma in a *M. marinum* wild type infected tail. Center contains acellular necrosis. Lower panel: High magnification of region with acellular necrosis, which is defined by an acellular, amorphous eosinophilic material centrally located in an inflammatory lesion.
(4.71 MB TIF)

Figure S7 Esx-1 negatively affects T cell infiltration into granulomatous lesions. CD3-positive cells in lesions in the tails of wild type and ΔRD1 infected B6 mice were counted as described in Methods. At least 3 lesions in 2 separate tails from each group were analyzed at each time point. Statistical significance was calculated by the Student's t-test (* *P*<0.05, **P<0.01).
(0.29 MB TIF)

Figure S8 Wild type and Esx-1-deficient *M. marinum* are similarly able to persist with minimal pathology in infected tails. B6 mice were infected with 1×10^7^ wild type and ΔRD1 bacteria, respectively, via tail vein injection. (A) CFU-analysis of the tails from 2 mice (Mouse #1 and #2) per group 76 days post infection indicated similar bacterial burdens in both wild type and ΔRD1 infected animals. (B) Similar analysis of bacterial burdens in tail tissues of wild type and ΔRD1 infected mice 120 days post infection. Values represent mean ± SD of three mice per group. (C) Quantification of the accumulated length (in cm) of all visible lesions in individual tails of wild type and ΔRD1 infected mice at indicated times post infection. Values represent mean ± SD of five mice per group.
(0.53 MB TIF)

Figure S9 Esx-1 promotes phagosome escape in vivo. High resolution captures from TEM analysis of infected cells in lesions in wild type (upper panel) and ΔRD1 (lower panel) infected tails. Intraphagosomal bacteria are indicated with an encircled asterix, and cytosolic bacteria with an asterix. Red arrows point to membranes of bacteria-containing vesicles. Wild type *M. marinum* was primarily found without an apparent surrounding vacuolar membrane, suggesting cytosolic localization. In contrast, virtually all ΔRD1 bacteria were observed within membraneous vesicles.
(6.01 MB TIF)

Figure S10 Esx-1 is required for IL-1β and IL-18 secretion in bone marrow-derived macrophages. Macrophages were infected as indicated. Supernatants were analyzed for IL-1β (left panel) and IL-18 (right panel) by Luminex 12 hrs post infection.
(0.38 MB TIF)

Figure S11 *M. marinum* induces TNFα and IL-6 secretion, but represses IL-12p40 secretion, in an Esx-1-dependent manner. Bone marrow-derived macrophages were infected with wild type or ΔRD1 bacteria as indicated, and analyzed for secretion of TNFα, IL-6 and IL-12p40 by Luminex 12 hrs post infection. Uninfected B6 macrophages were analyzed as control. Shown are data for at least three separate experiments. Statistical analysis (Student's t-test; *P<0.05, **P<0.01) indicated a significant Esx-1-dependent regulation of all 3 cytokines; samples with smallest difference between the two groups (wild type and ΔRD1 infected cells) compared.
(0.55 MB TIF)
