# Supplementary figures and images for: Host-Detrimental Role of Esx-1-Mediated Inflammasome Activation in Mycobacterial Infection
Source: PLoS Pathog. 2010 May 6;6(5):e1000895. doi: 10.1371/journal.ppat.1000895 (PMC2865529; doi:10.1371/journal.ppat.1000895)

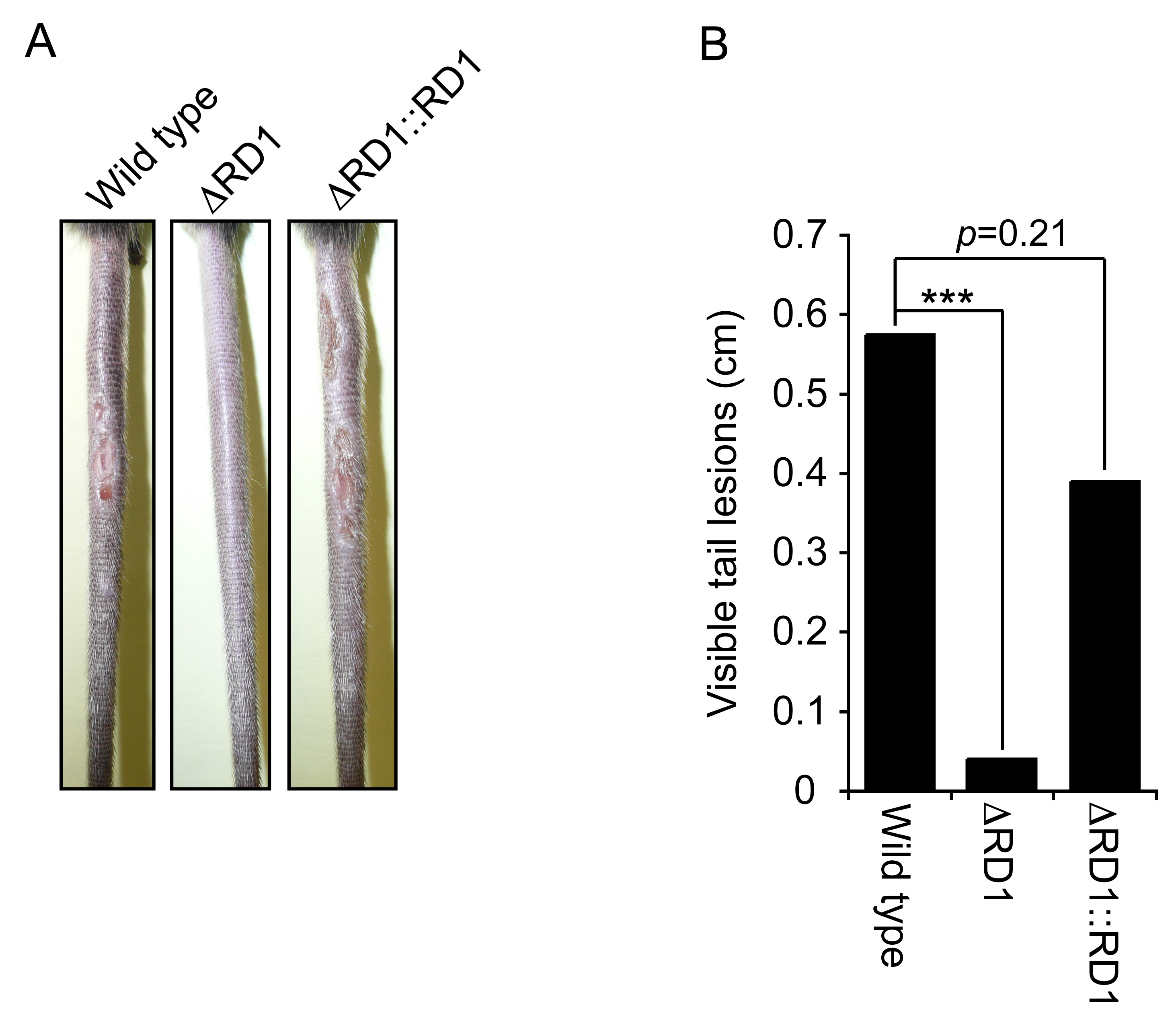

Supplement: Figure S1 — Complementation of M. marinum ΔRD1 bacteria with the M. tuberculosis-derived RD1-locus restores ability to cause disease. B6 mice were infected with 1×10^7^ wild type, ΔRD1 or ΔRD1::RD1 bacteria via tail vein injection, as indicated. (A) Shown is representative tails 15 days post infection. (B) Quantification of the accumulated length (in cm) of all visible lesions in individual tails of wild type, ΔRD1 and ΔRD1::RD1 infected mice at 15 days post infection. Values represent mean of 10 mice per group. Statistical significance was calculated by the Student's t-test (* P<0.05, **P<0.01, ***P<0.001). (3.61 MB TIF) [file ppat.1000895.s001.tif]

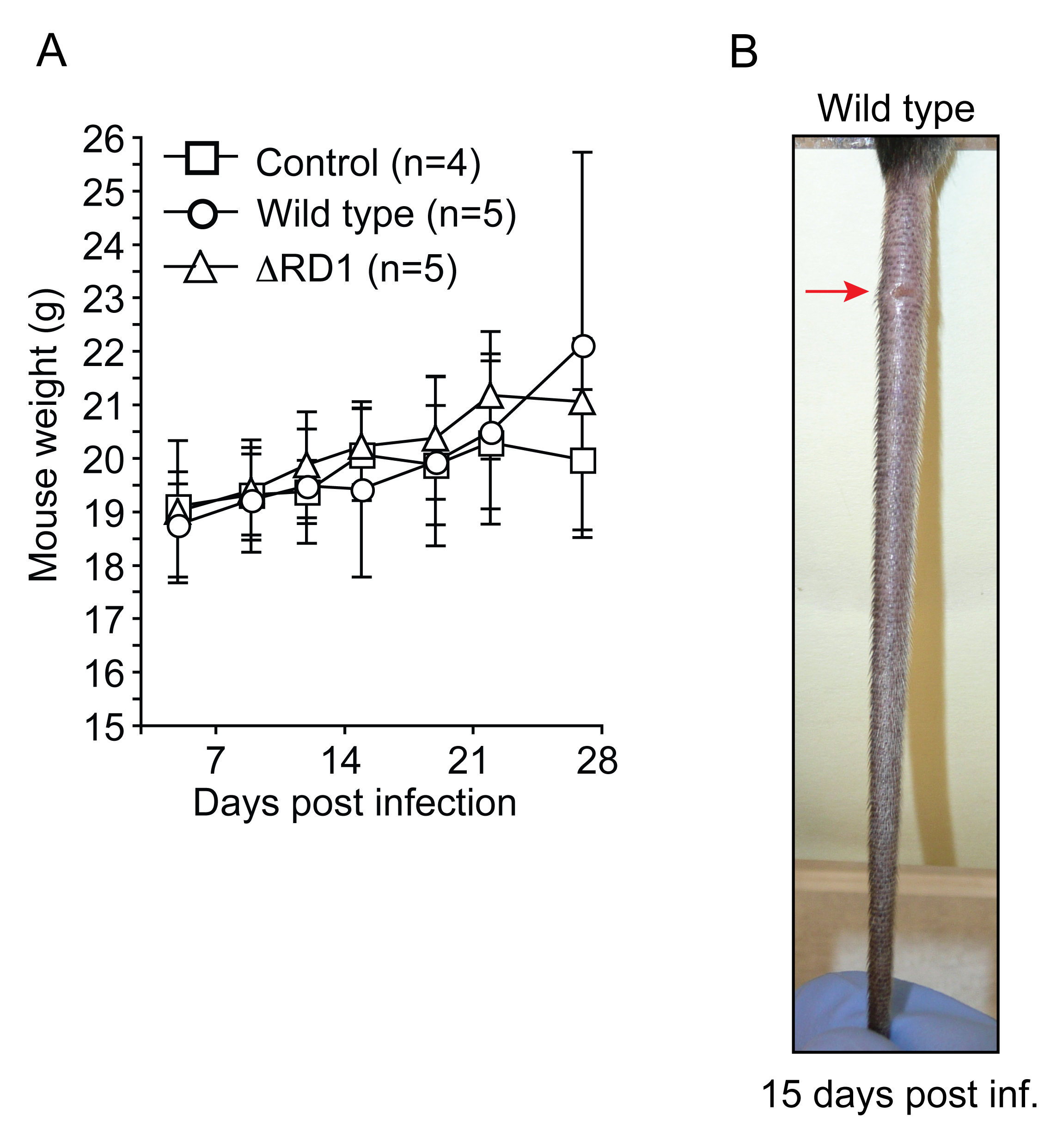

Supplement: Figure S2 — M. marinum cause local disease in the tail. (A) B6 mice were infected with 1×10^7^ bacteria via tail vein injection as indicated, and monitored for weight changes. Weight development was unaffected by infection, suggesting that M. marinum does not cause significant systemic effects. Control mice were similarly injected with PBS. (B) B6 mice were infected with 1×10^7^ wild type M. marinum via intra cardiac injection and followed over time for appearance of lesions. Lesions (indicated with red arrow) were observed in the tail of infected animals ∼15 days post infection, suggesting that the bacteria spread via the blood and specifically established an infection in the tail. (3.01 MB TIF) [file ppat.1000895.s002.tif]

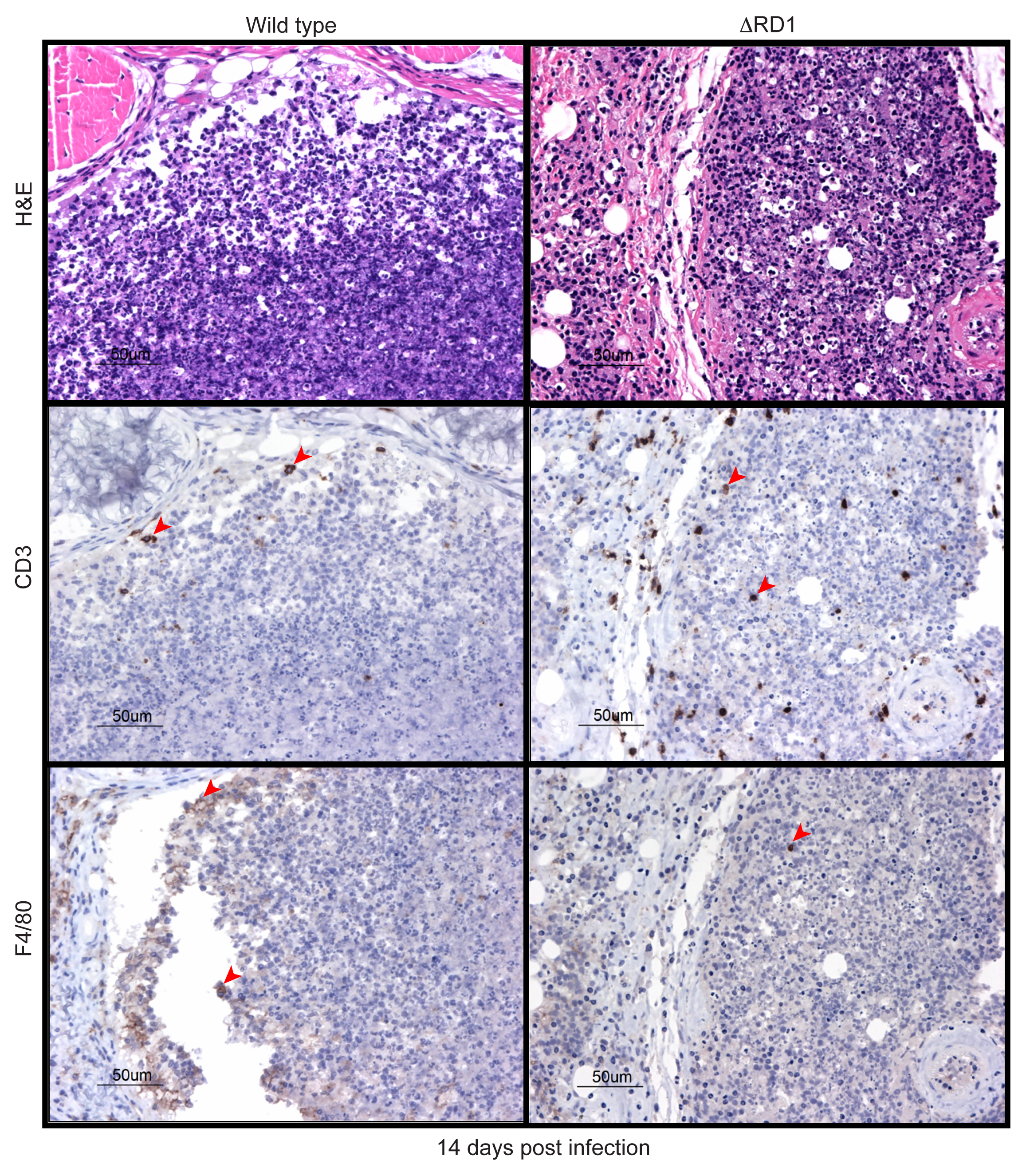

Supplement: Figure S3 — Histological analysis of granulomas 14 days post infection. High magnification of data presented in Figure 2A. For clarity, examples of immunostained cells are indicated with red arrowheads. (9.43 MB TIF) [file ppat.1000895.s003.tif]

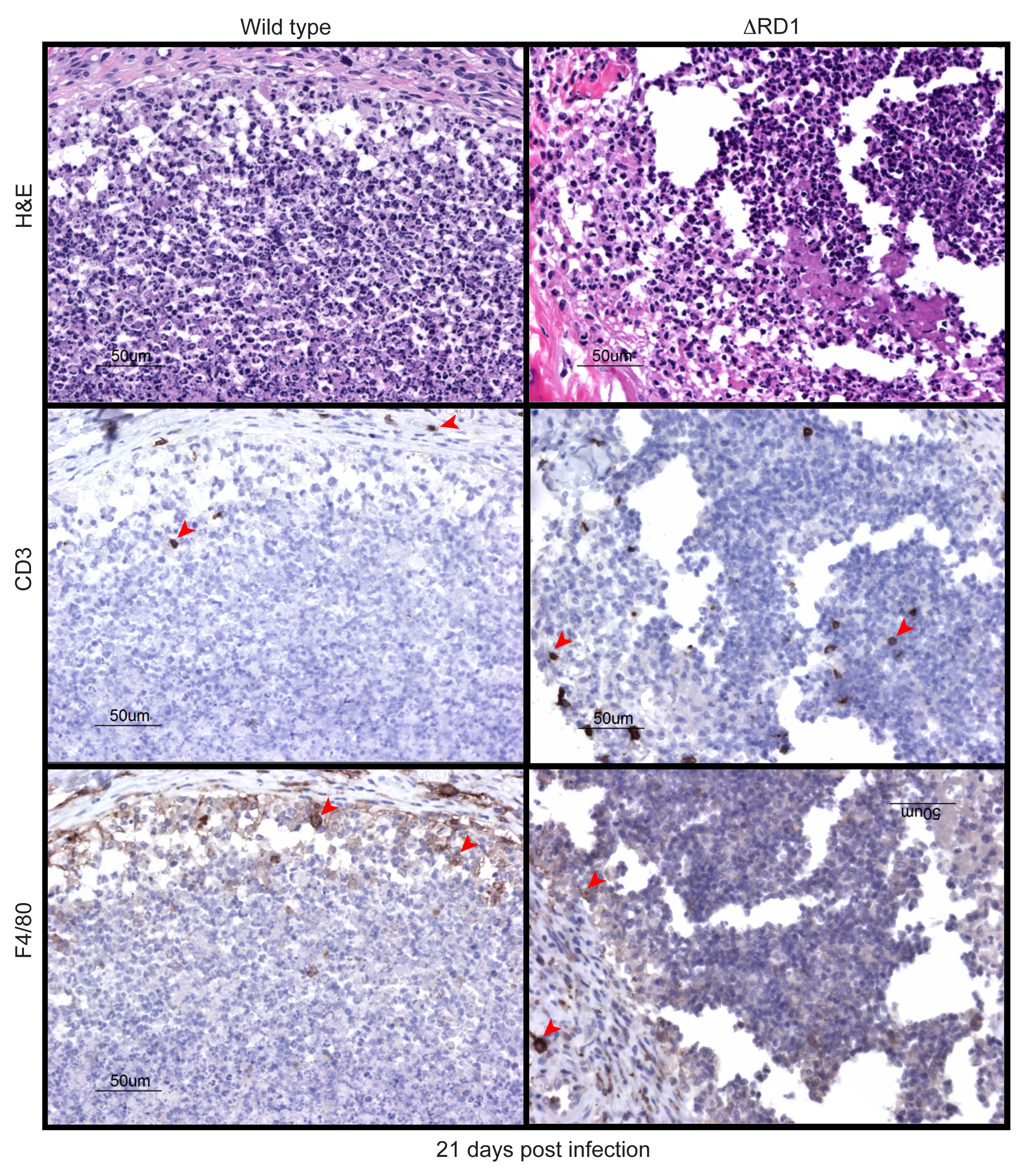

Supplement: Figure S4 — Histological analysis of granulomas 21 days post infection. High magnification of data presented in Figure 2B. For clarity, examples of immunostained cells are indicated with red arrowheads. (8.85 MB TIF) [file ppat.1000895.s004.tif]

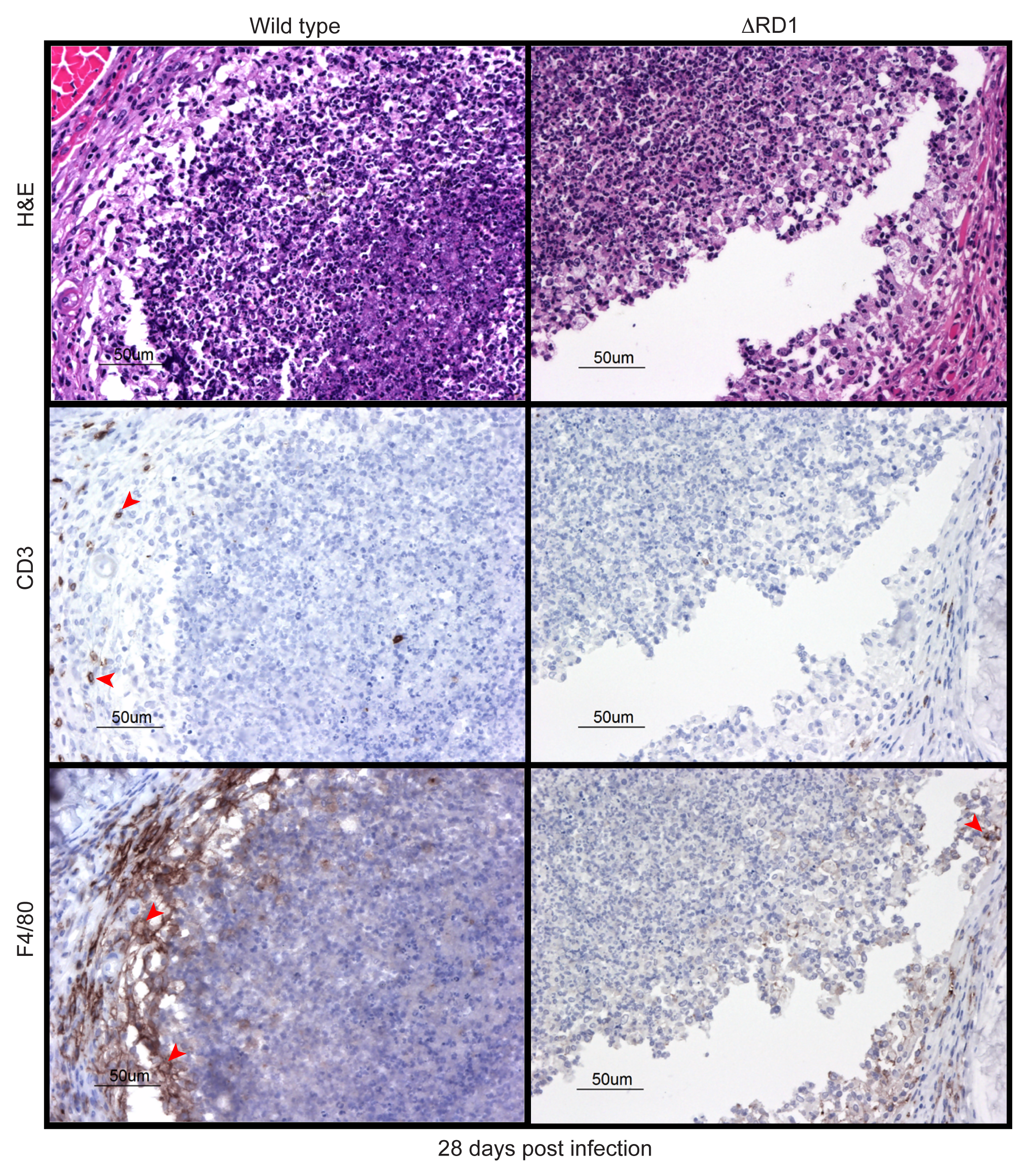

Supplement: Figure S5 — Histological analysis of granulomas 28 days post infection. High magnification of data presented in Figure 2C. For clarity, examples of immunostained cells are indicated with red arrowheads. (9.19 MB TIF) [file ppat.1000895.s005.tif]

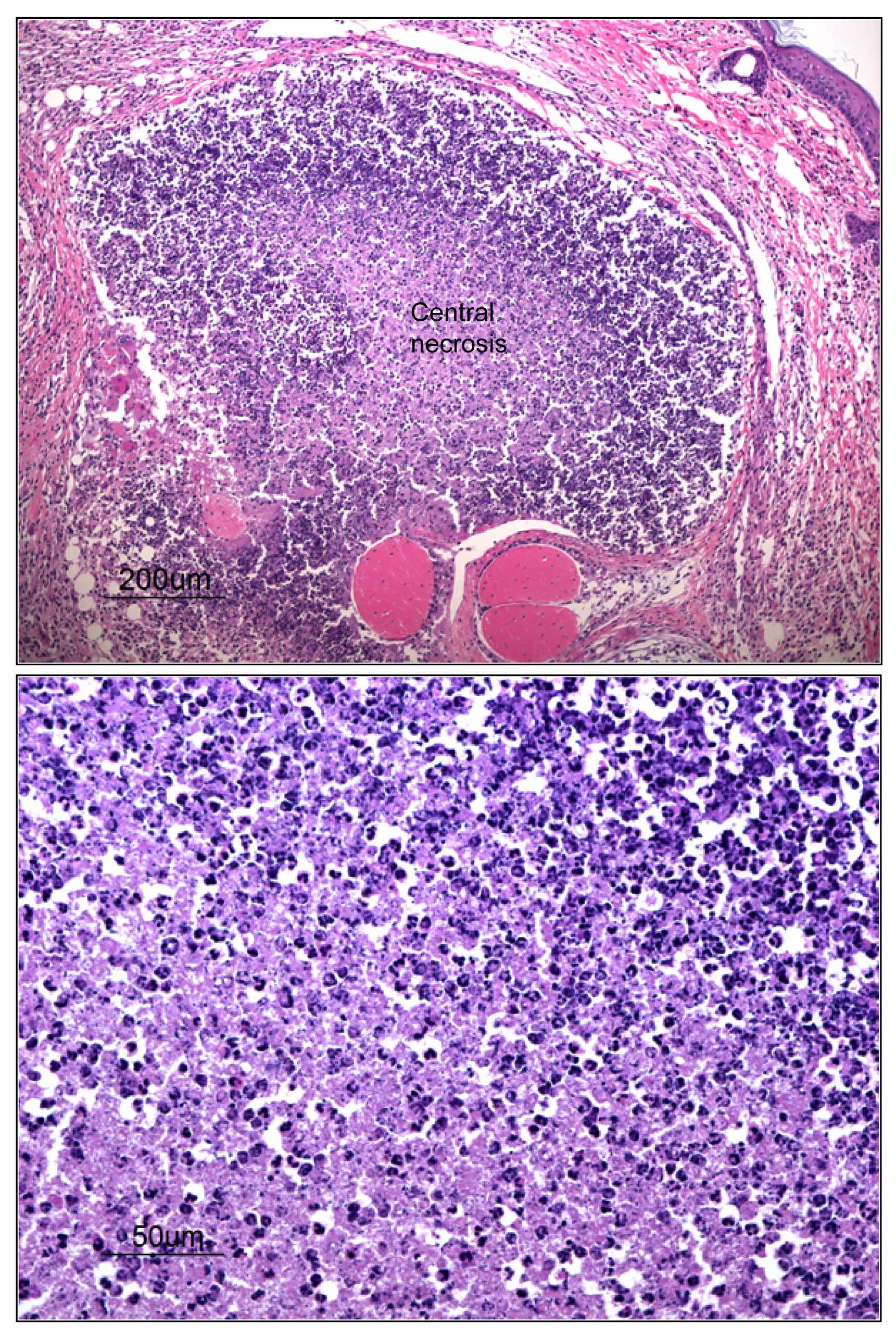

Supplement: Figure S6 — Caseating centers in M. marinum wild type induced granulomas. High magnification of data presented in Figure 2D. Upper panel: H&E staining of a granuloma in a M. marinum wild type infected tail. Center contains acellular necrosis. Lower panel: High magnification of region with acellular necrosis, which is defined by an acellular, amorphous eosinophilic material centrally located in an inflammatory lesion. (4.71 MB TIF) [file ppat.1000895.s006.tif]

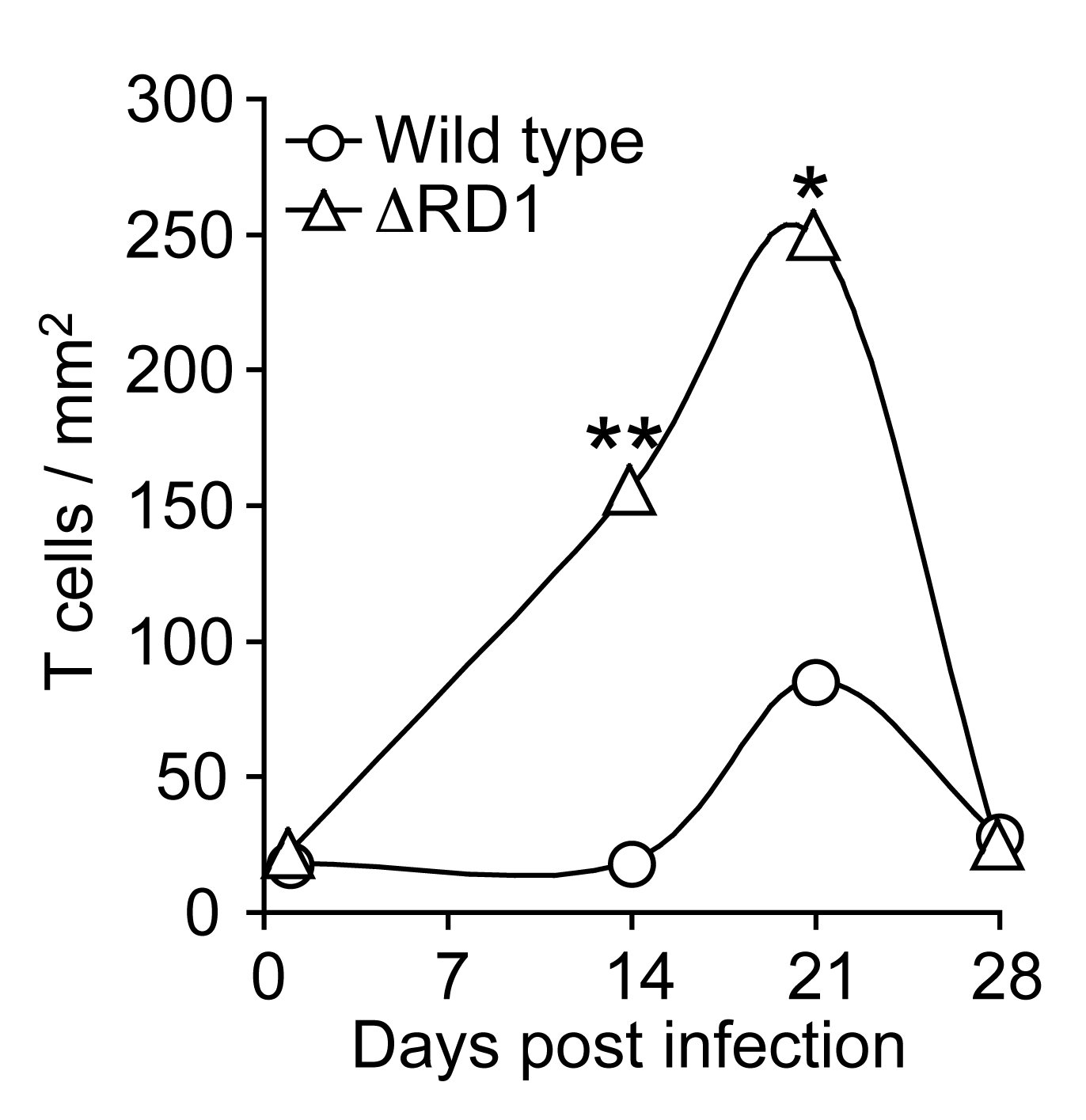

Supplement: Figure S7 — Esx-1 negatively affects T cell infiltration into granulomatous lesions. CD3-positive cells in lesions in the tails of wild type and ΔRD1 infected B6 mice were counted as described in Methods. At least 3 lesions in 2 separate tails from each group were analyzed at each time point. Statistical significance was calculated by the Student's t-test (* P<0.05, **P<0.01). (0.29 MB TIF) [file ppat.1000895.s007.tif]

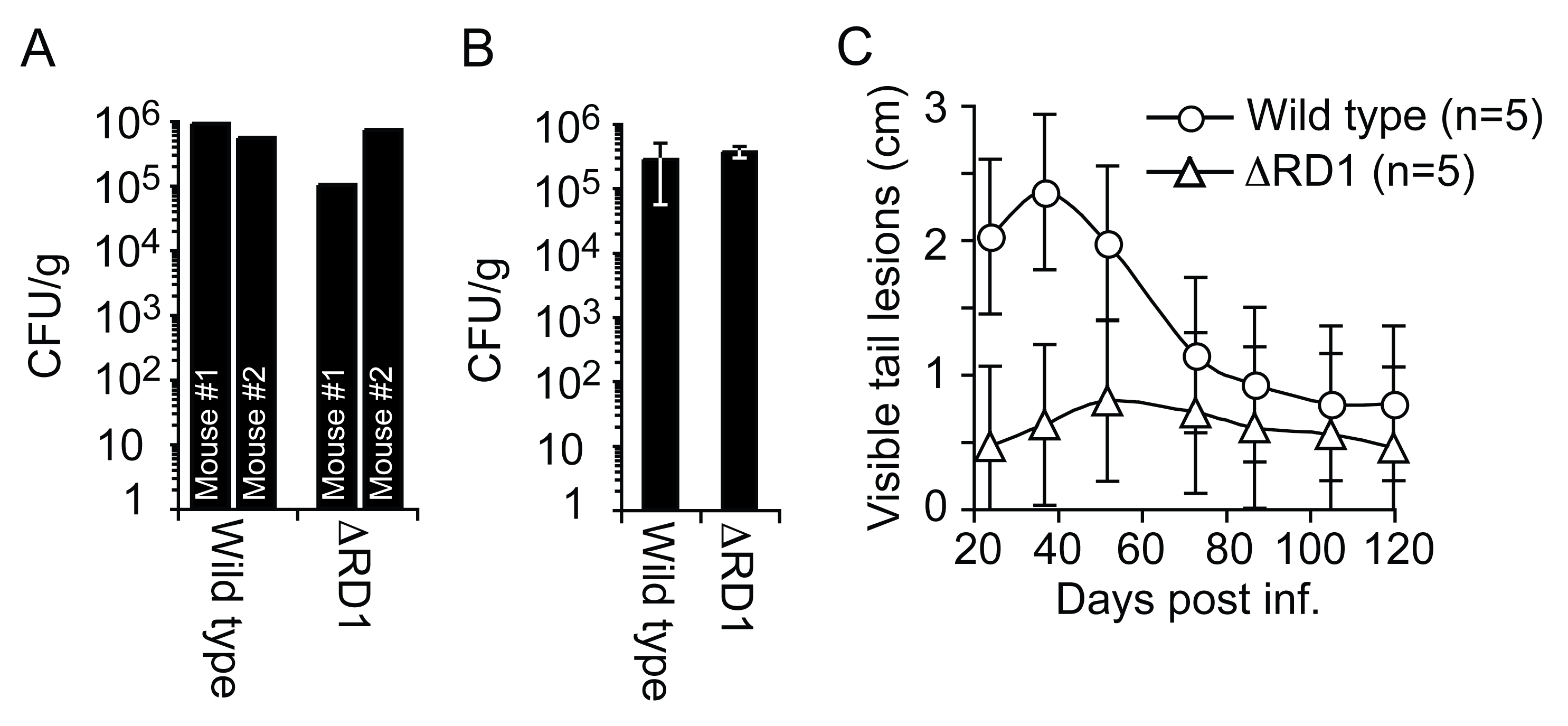

Supplement: Figure S8 — Wild type and Esx-1-deficient M. marinum are similarly able to persist with minimal pathology in infected tails. B6 mice were infected with 1×10^7^ wild type and ΔRD1 bacteria, respectively, via tail vein injection. (A) CFU-analysis of the tails from 2 mice (Mouse #1 and #2) per group 76 days post infection indicated similar bacterial burdens in both wild type and ΔRD1 infected animals. (B) Similar analysis of bacterial burdens in tail tissues of wild type and ΔRD1 infected mice 120 days post infection. Values represent mean ± SD of three mice per group. (C) Quantification of the accumulated length (in cm) of all visible lesions in individual tails of wild type and ΔRD1 infected mice at indicated times post infection. Values represent mean ± SD of five mice per group. (0.53 MB TIF) [file ppat.1000895.s008.tif]

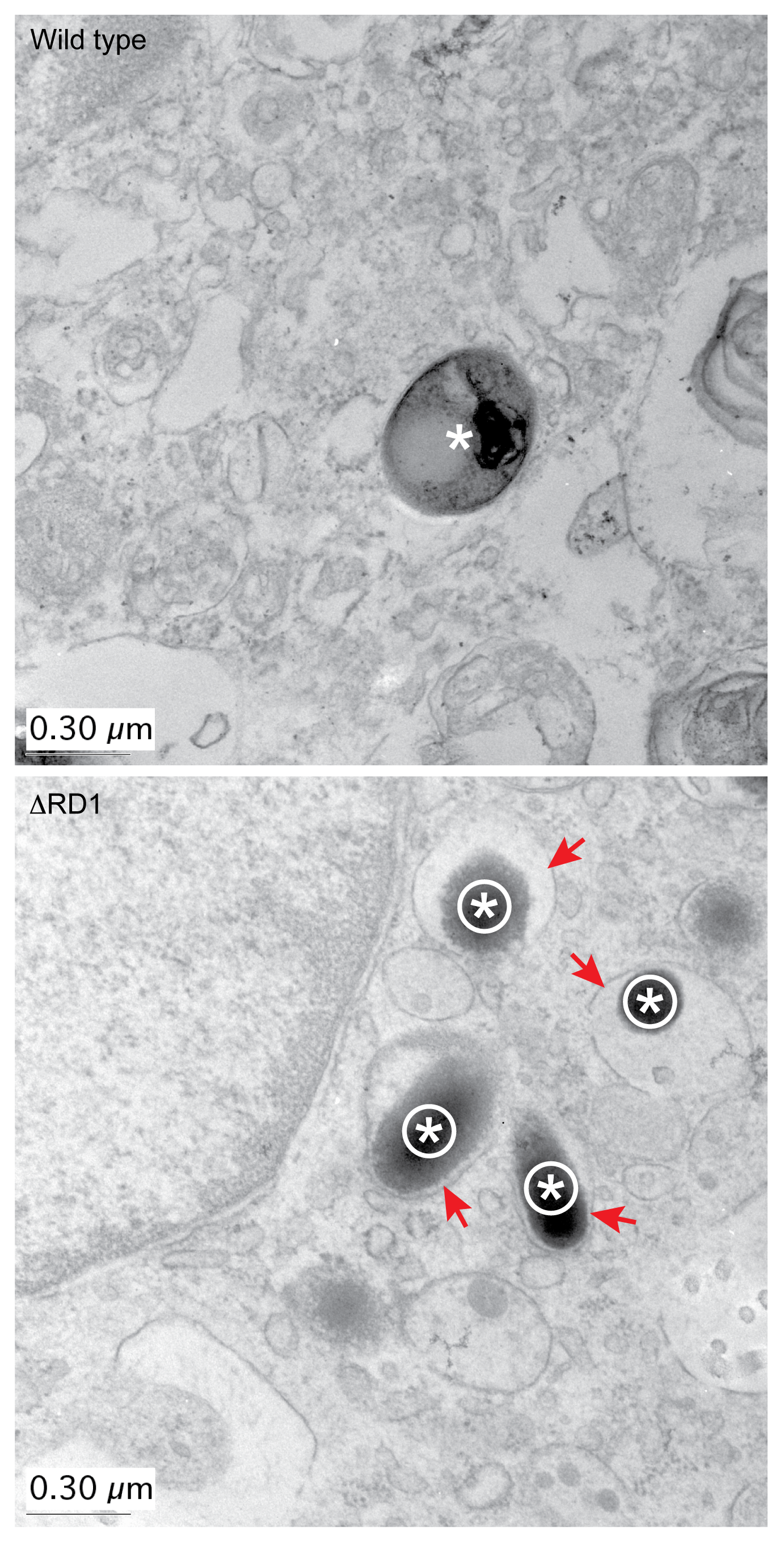

Supplement: Figure S9 — Esx-1 promotes phagosome escape in vivo. High resolution captures from TEM analysis of infected cells in lesions in wild type (upper panel) and ΔRD1 (lower panel) infected tails. Intraphagosomal bacteria are indicated with an encircled asterix, and cytosolic bacteria with an asterix. Red arrows point to membranes of bacteria-containing vesicles. Wild type M. marinum was primarily found without an apparent surrounding vacuolar membrane, suggesting cytosolic localization. In contrast, virtually all ΔRD1 bacteria were observed within membraneous vesicles. (6.01 MB TIF) [file ppat.1000895.s009.tif]

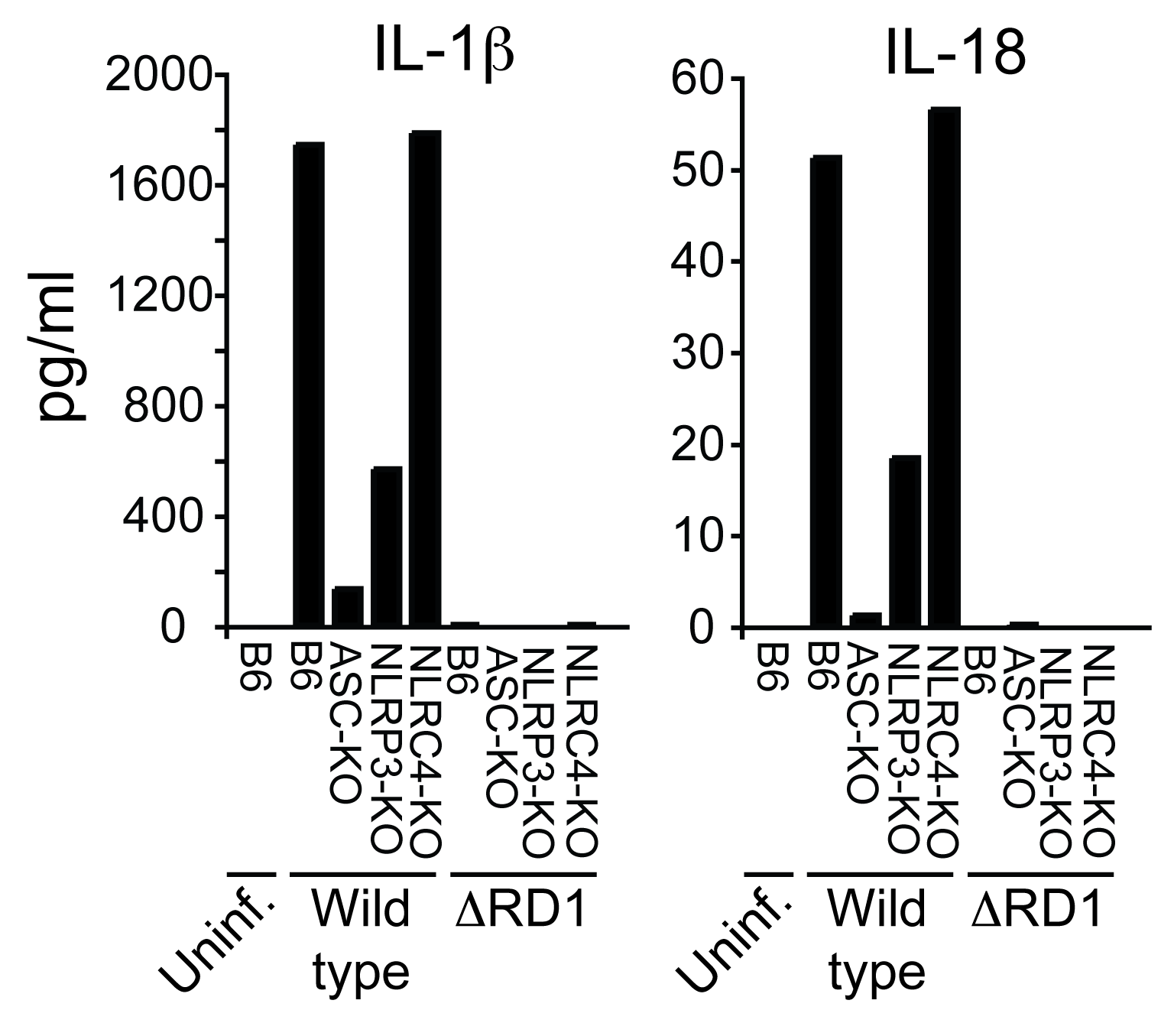

Supplement: Figure S10 — Esx-1 is required for IL-1β and IL-18 secretion in bone marrow-derived macrophages. Macrophages were infected as indicated. Supernatants were analyzed for IL-1β (left panel) and IL-18 (right panel) by Luminex 12 hrs post infection. (0.38 MB TIF) [file ppat.1000895.s010.tif]

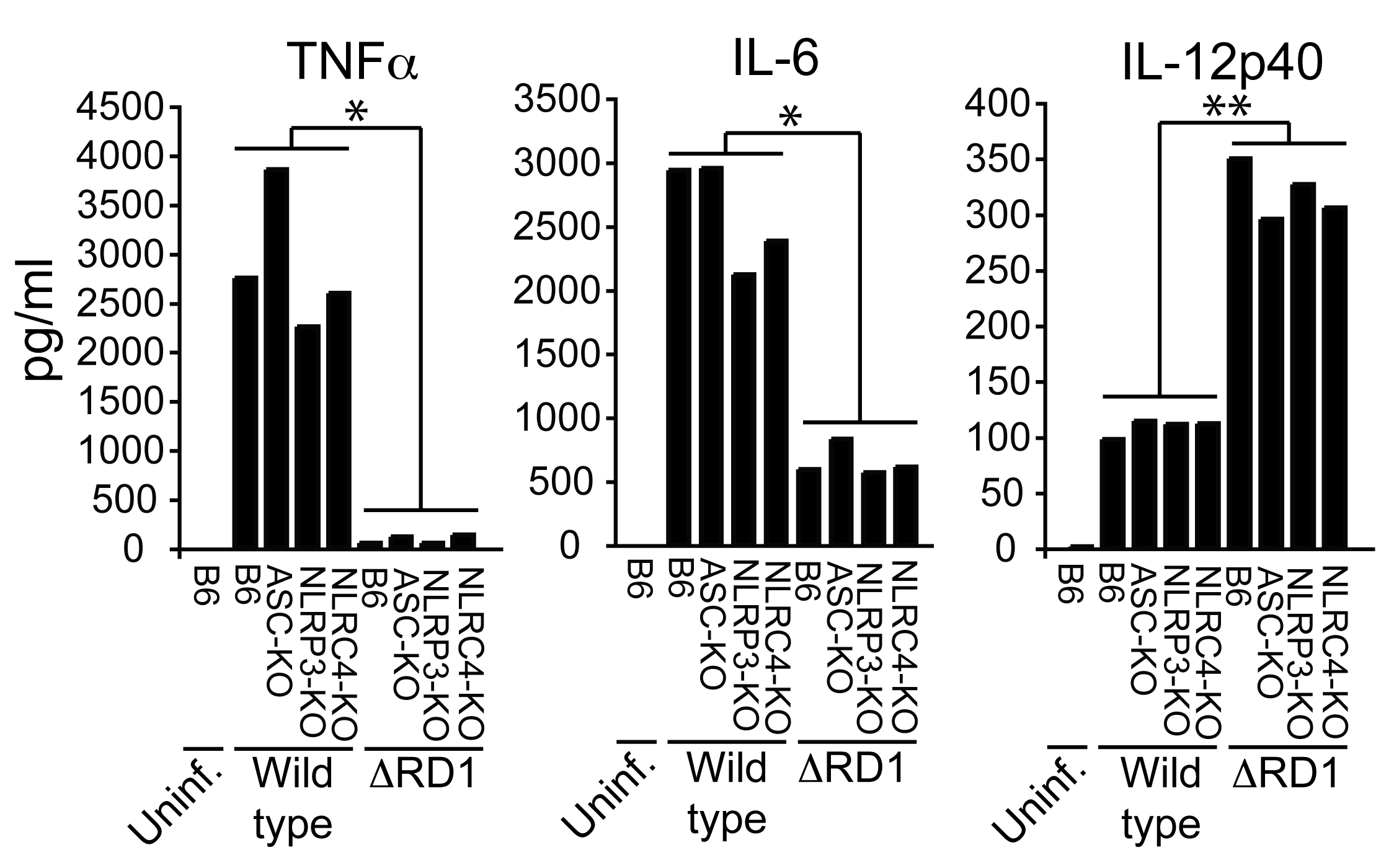

Supplement: Figure S11 — M. marinum induces TNFα and IL-6 secretion, but represses IL-12p40 secretion, in an Esx-1-dependent manner. Bone marrow-derived macrophages were infected with wild type or ΔRD1 bacteria as indicated, and analyzed for secretion of TNFα, IL-6 and IL-12p40 by Luminex 12 hrs post infection. Uninfected B6 macrophages were analyzed as control. Shown are data for at least three separate experiments. Statistical analysis (Student's t-test; *P<0.05, **P<0.01) indicated a significant Esx-1-dependent regulation of all 3 cytokines; samples with smallest difference between the two groups (wild type and ΔRD1 infected cells) compared. (0.55 MB TIF) [file ppat.1000895.s011.tif]
